# Quorum sensing in thermophiles: prevalence of autoinducer-2 system

**DOI:** 10.1186/s12866-018-1204-x

**Published:** 2018-06-28

**Authors:** Amandeep Kaur, Neena Capalash, Prince Sharma

**Affiliations:** 1 Department of Microbiology, Panjab University, Chandigarh, India; 2 Department of Biotechnology, Panjab University, Chandigarh, India

**Keywords:** Thermophiles, Quorum sensing, Autoinducer-2, LuxS, LsrB, RbsB, SAH hydrolase

## Abstract

**Background:**

Quorum sensing is a mechanism of cell to cell communication that requires the production and detection of signaling molecules called autoinducers. Although mesophilic bacteria is known to utilize this for synchronization of physiological processes such as bioluminescence, virulence, biofilm formation, motility and cell competency through signaling molecules (acyl homoserine lactones, AI-1; oligopeptides, peptide based system and furanosyl borate diester, AI-2), the phenomenon of quorum sensing in thermophiles is largely unknown.

**Results:**

In this study, proteomes of 106 thermophilic eubacteria and 21 thermophilic archaea have been investigated for the above three major quorum sensing systems to find the existence of quorum sensing in these thermophiles as there are evidences for the formation of biofilms in hot environments. Our investigation demonstrated that AI-1 system is absent in thermophiles. Further, complete peptide based two component systems for quorum sensing was also not found in any thermophile however the traces for the presence of response regulators for peptide based system were found in some of them. BLASTp search using LuxS (AI-2 synthase) protein sequence of *Escherichia coli* str. K-12 substr. MG1655 and autoinducer-2 receptors (LuxP of *Vibrio harveyi*, LsrB of *E. coli* str. K-12 substr. MG1655 and RbsB of *Aggregatibacter actinomycetemcomitans*) as queries revealed that 17 thermophilic bacteria from phyla *Deinococcus- Thermus* and *Firmicutes* possess complete AI-2 system (LuxS and LsrB and/or RbsB). Out of 106 thermophilic eubacteria 18 from phyla *Deinococcus- Thermus*, *Proteobacteria* and *Firmicutes* have only LuxS that might function as AI-2 synthesizing protein whereas, 16 are having only LsrB and/or RbsB which may function as AI-2 receptor in biofilms.

**Conclusions:**

We anticipate that thermophilic bacteria may use elements of LsrB and RbsB operon for AI-2 signal transduction and they may use quorum sensing for purposes like biofilm formation. Nevertheless, thermophiles in which no known quorum sensing system was found may use some unknown mechanisms as the mode of communication. Further information regarding quorum sensing will be explored to develop strategies to disrupt the biofilms of thermophiles.

**Electronic supplementary material:**

The online version of this article (10.1186/s12866-018-1204-x) contains supplementary material, which is available to authorized users.

## Background

Quorum sensing is a cell density dependent mode of communication used by bacteria to sense their neighbors and allows them to synchronize their behavior. In mesophilic bacteria, quorum sensing is known to regulate various collaborative processes viz. virulence, bioluminescence, competence, biofilm formation, swarming, sporulation, motility, regulation of stress related genes, optimal growth under iron starvation and antibiotic resistance. These processes are controlled by the production and detection of signaling molecules termed as autoinducers [1–5]. Despite such vast information regarding quorum sensing in mesophiles, the information in thermophiles is largely unknown, although the processes like formation of biofilms by thermophilic bacteria and archaea are well recognized [6–8].

Although the three major quorum sensing systems (Autoinducer-1, Peptide based and Autoinducer-2) have been investigated in thermophilic bacteria but still there is limited information on the mechanism of quorum sensing in thermophilic environment [9]. Autoinducer-1 (acyl homoserine lactone, AHLs) is known to be heat labile, so this kind of quorum sensing was thought unlikely at high temperature [10]. Peptide based quorum sensing has been observed in hyperthermophilic syntrophic cultures like the association between *Thermotoga maritima,* which produces H_2_ as autoinhibitory product and hydrogenotrophic methanogen *Methanococcus jannaschii* [7]. Here, the inhibition of growth of *T. maritima* due to H_2_ is relieved and cell density is enhanced by transfer of H_2_ to *M. jannaschii.* Autoinducer-2 (AI-2) is known to be a universal system of bacterial communication. Nichols et al. [11] demonstrated that two hyperthermophiles, a eubacterium *T. maritima*, and an archaean *Pyrococcus furiosus*, produced a response to *Vibrio harveyi* AI-2 assay despite lacking the autoinducer-2 synthase LuxS (also known as S-ribosyl homocysteine lyase or S-ribosylhomocysteinase). They further hypothesized that temperature dependent rearrangement of phosphorylated ribose results in formation of AI-2 in these hyperthermophiles. Perez-Rodriguez et al. [12] demonstrated that the *luxS* is expressed and quorum sensing is induced in *Caminibacter mediatlanticus*, an epsilon proteobacterium from deep-sea hydrothermal vents. They further represented the evolutionary linkage of *luxS* genes among the thermophiles and human pathogens of epsilon proteobacteria. Rao et al., [13] demonstrated the presence of LuxS homologues in certain thermophilic members of *Deinococcus-Thermus* and *Firmicutes*. However, to date, no report exists on the exploration of complete autoinducer-2 pathway comprising autoinducer-2, methylthioadenosine/S-adenosylhomocysteine nucleosidase (also known as MTA/SAH nucleosidase or Pfs) and autoinducer-2 receptor in thermophilic bacteria.

**Fig. 1:**
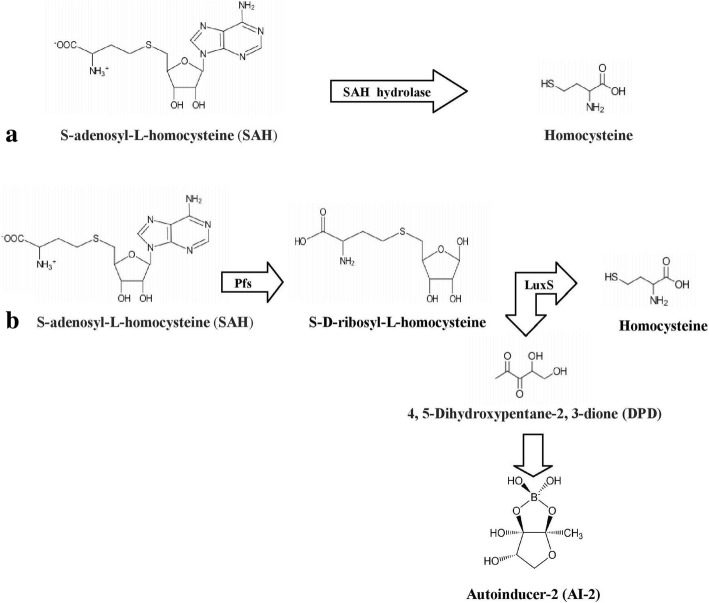
Pathways for detoxification of SAH **a** One step pathway **b** Two step pathway

LuxS is known to be involved in quorum sensing as well as in activated methyl cycle (AMC). Quorum sensing pathway involves two steps, the conversion of toxic S-adenosyl-homocysteine to S-ribosyl-homocysteine catalyzed by Pfs and then conversion of S-ribosyl-homocysteine to homocysteine and 4,5-dihydroxy-2,3-pentanedione (DPD), catalyzed by LuxS. This DPD is further converted to AI-2 (a furanosyl borate diester) (Fig. 1).

This study is aimed at assessing which type(s) of quorum sensing system(s) is prevalent in thermophiles. For this, we have investigated all the three major quorum sensing systems (Autoinducer-1, Peptide based system and Autoinducer-2) in 106 thermophilic eubacteria and 21 thermophilic archaea. Through extensive database analysis of their proteomes, it was found that no autoinducer-1 like synthase or receptor is present in thermophiles. However traces of the presence of peptide based system have been found in certain thermophilic eubacteria. This study also investigates AI-2 synthase (LuxS), Pfs as well as receptors for autoinducer-2 (LuxP, LsrB and RbsB) by analyzing proteomes of thermophilic bacteria and thermophilic archaea in NCBI. These finding provide new insights into the mode of communication utilized by thermophilies (planktonic/biofilms) at high temperature.

## Results

### Prevalence of quorum sensing systems in thermophiles

The proteomes of 106 thermophilic eubacteria and 21 thermophilic archaea were searched for the presence of autoinducer-1 system through BLASTp using LuxI and LuxR protein sequences of *V. fischeri* ES114 as query. Not more than 30% identity with 40% query cover was found in any of the thermophiles. None of the thermophiles was found to have autoinducer-1 system as it is already known that AHLs are degraded at high temperature [10].

Peptide based quorum sensing was searched through Quorumpeps software which comprises all the known quorum sensing signaling peptides [14]. *T. maritima* which is already reported [7] to exhibit quorum sensing through peptide signaling was the only thermophile found in this study. Next BLASTp search was performed for finding the peptide based signaling in other thermophiles by using protein sequences of five peptide based quorum sensing systems (Additional file 1) as query but very low (less than 35%) identity was found in any of the thermophilic eubacteria (Additional file 2, Additional files 3, 4 and 5). Among the five peptide based sequences used for BLASTp, none of the histidine kinase receptors showed identity with thermophiles while few thermophilic eubacteria had query cover more than 90% and identity more than 28% with response regulator AgrA and ComA (Additional file 2), and MultAlin *s*howed very little conservation (Additional files 3, 4 and 5). None of the thermophilic archaea showed identity with any of the protein sequences (Additional file 1).

### Prevalence of enzymes involved in synthesis of DPD

#### Pfs and LuxS

A total of 106 thermophilic eubacteria were used for the analysis. As shown in Table 2, 40 were having LuxS protein showing identity (36 to 66%) with LuxS of *E. coli* str. K-12 substr. MG1655. *Nitratiruptor* sp. SB155–2 was found to have maximum identity (66%) while *Anoxybacillus spp.* had minimum (36–39%). Further, to complete the analysis for the presence of quorum sensing in thermophiles, search for the presence of LuxS in archaea was performed, but it was not found in any archean (Table 1).

**Table 1:** Exploring the presence of LuxS, Pfs, LuxP, LsrB, RbsB and SAH hydrolase in thermophililc archaea by using the protein sequences of LuxS, Pfs, and LsrB of *E. coli* str. K-12 substr. MG1655, LuxP of *V. harveyi,* RbsB of *A. actinomycetemcomitans* and SAH hydrolase of *Ralstonia solanacearum* as queries

S.No.	Phylum	Archaea	Growth Temperature (°C)	A1–1 system	A1–2 system (% identity)					
					LuxS	LuxP	LsrB	RbsB	Pfs	SAH hydrolase
1.	Euryarchaeota	*Thermococcus barophilus*	(70- < 100)	–	–	–	–	–	–	40
		*Thermococcus celer*		–	–	–	–	–	–	–
		*Thermococcus chitonophagus*		–	–	–	–	–	–	41
		*Thermococcus gammatolerans*		–	–	–	–	–	–	41
		*Thermococcus hydrothermalis*		–	–	–	–	–	–	–
		*Thermococcus kodakarensis*		–	–	–	–	–	–	41
		*Thermococcus litoralis*		–	–	–	–	–	–	42
		*Thermococcus profundus*		–	–	–	–	–	–	–
		*Thermococcus stetteri*		–	–	–	–	–	–	–
		*Pyrococcus abyssi*	100	–	–	–	–	–	–	41
		*Pyrococcus furiosus*		–	–	–	–	–	–	42
		*Pyrococcus horikoshii*		–	–	–	–	–	–	41
		*Pyrococcus woesei*		–	–	–	–	–	–	–
		*Picrophilus oshimae*	60	–	–	–	–	–	–	–
		*Picrophilus torridus*		–	–	–	–	–	–	41
2.	Crenarchaeota	*Sulfolobus shibatae*	80	–	–	–	–	–	–	–
		*Pyrolobus fumarii*	113	–	–	–	–	–	–	43
		*Pyrodictium abyssi*	110	–	–	–	–	–	–	–
		*Metallosphaera sedula*	75	–	–	–	–	–	–	41
		*Aeropyrum pernix*	(70–100)	–	–	–	–	–	30	42
3.	Nanoarchaeota	*Nanoarchaeum equitans*	80	–	–	–	–	–	–	–

^a^+: Present

^b^-: < 25% identity not present

The LuxS sequences of thermophlic bacteria were aligned with those of mesophiles to find the conservation of residues. The “invariant sequence motif”, HXXEH was found in all these bacteria besides amino acids viz. Ser-9, Phe-10, Asp-13, His-14, Ala-19, Pro-20, Val-22, Arg-23, Asp-40, Arg-43, Pro-46, Asn-47, Ala-64, Arg-68, residues 80–95, Gly-97, Pro-125, Cys-132, Gly-133, His-138, Ala-143 and Leu-151 conserved in LuxS protein sequence of thermophilic as well as mesophilic bacteria (Additional file 6).

**Fig. 2:**
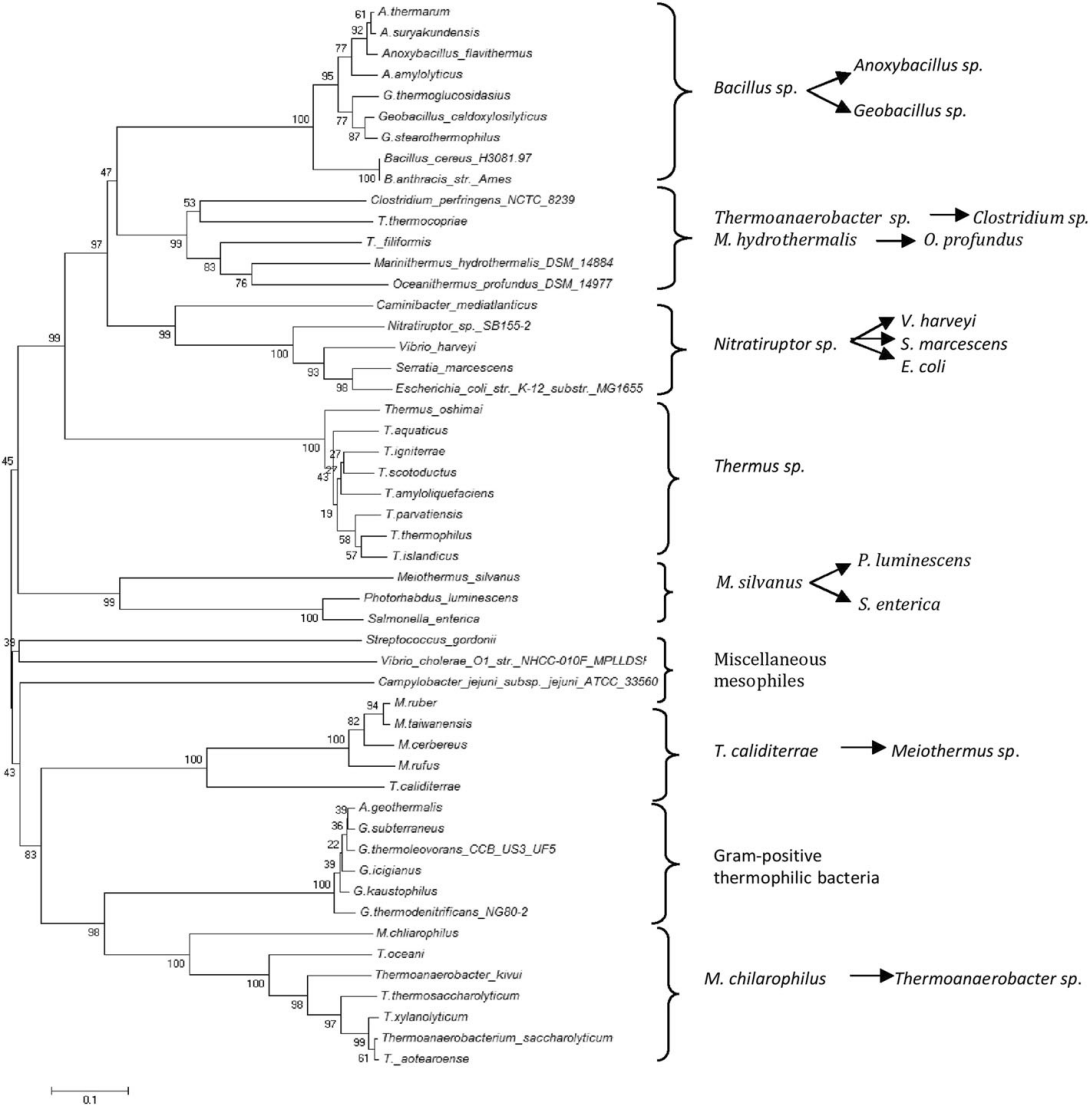
Evolutionary relationship of LuxS of thermophilic and mesophilc bacteria

**Fig. 3:**
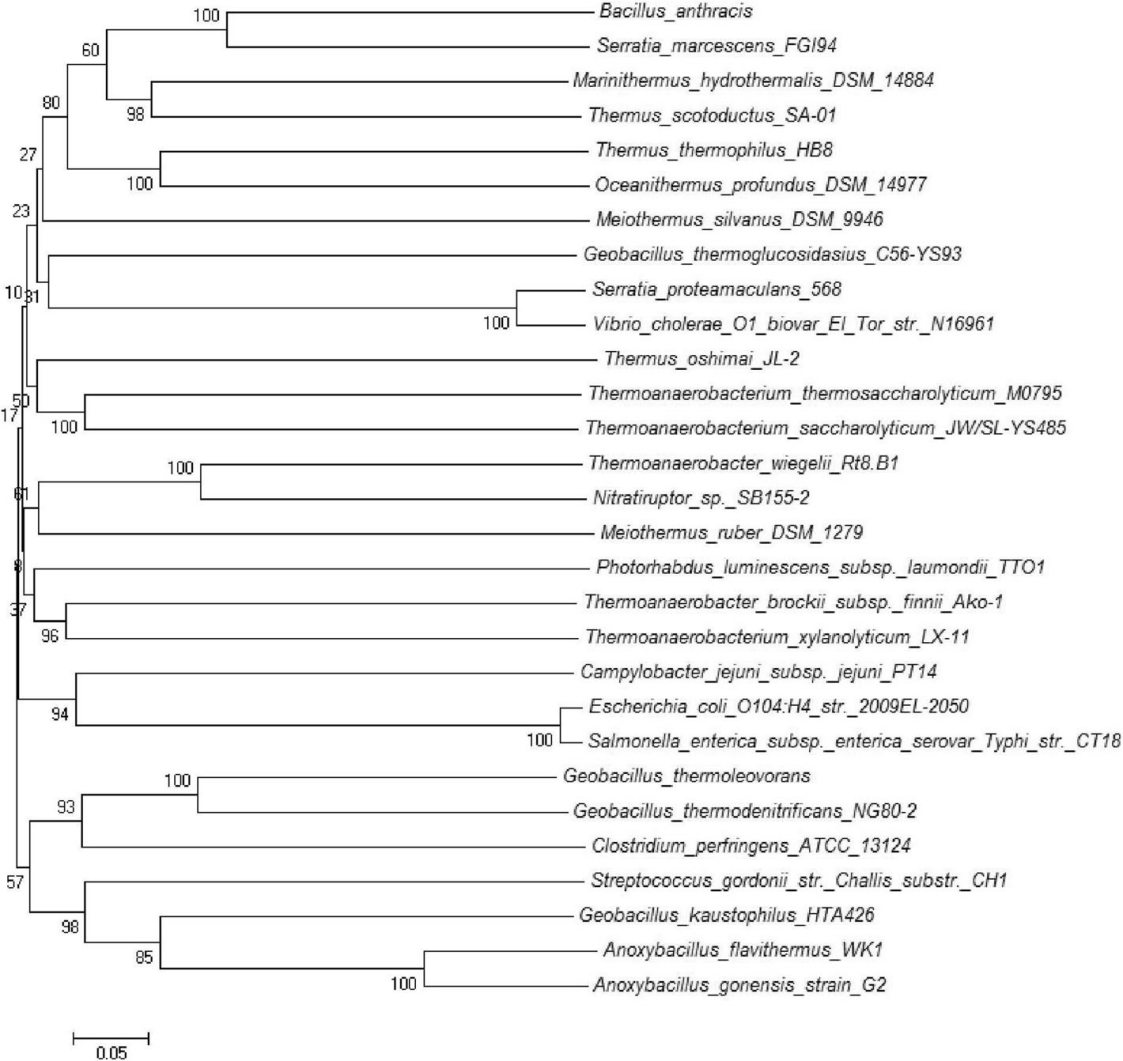
Evolutionary relationship of 16S rRNA of thermophilic and mesophilc bacteria having LuxS

Phylogenetic analysis of LuxS from mesophilic and thermophilic bacteria (Fig. 2) showed nine distinct clades formed. First clade comprising thermophilic Gram positive sporulating bacteria indicated that they shared a common *Bacillus spp.* (*B. cereus*/ *B. anthracis*) as ancestor. Second clade was formed by distinct thermophilic bacteria from different genera showing the evolutionary linkage between *T. thermocopriae* and *C. perfringens* as well as between *M. hydrothermalis* and *O. profundus*. *T. thermocopriae,* previously known as *Clostridium thermocopriae* and *C. perfringens* are Gram-positive anaerobic bacteria most commonly found in decaying vegetables. On the other hand *M. hydrothermalis* and *O. profundus* are deep-sea hydrothermal vent thermophiles. This information indicates that the bacteria prevalent in similar environments share relatedness among their LuxS sequences. In the third clade, *S. marcescens* and *E. coli* shared a common ancestor *V. harveyi* nested within the thermophilic lineages. They all belong to the phylum *Proteobacteria*. This suggests the thermophilic origin of LuxS among *Proteobacteria*. Fourth clade was formed by *Thermus spp.* with *T. oshimai* being the ancestor as all other LuxS of *Thermus spp*. are nested within it. Fifth clade showed evolutionary linkage of *P. luminescens* and *S. enterica* sharing a common thermophilic ancestor *M. silvanus.* This again suggested the thermophilic lineage of LuxS*.* Sixth clade comprised of mesophiles far distinct in evolutionary linkage*.* Seventh included *Meiothermus spp*., sharing *T. calditterrae* as ancestor. Eighth and ninth comprised of Gram positive thermophilic bacteria and *Thermoanaerobacter spp*. respectively*.* In ninth clade all *Thermoanaerobacteria spp*. were sharing a common ancestor (*M. chilarophilus*). LuxS was highly conserved among mesophiles and thermophiles while the conservation on the basis of 16S rRNA was not observed (Fig. 3).

The first enzyme that leads to detoxification of S-adenosylhomocysteine is MTA/SAH nucleosidase (Pfs), presence of which together with LuxS confirms the synthesis of autoinducer-2 precursor, DPD. Therefore, we searched for the presence of Pfs in thermophilic bacteria by using MTA/SAH nucleosidase sequence of *E. coli* str. K-12 substr as query (Table 2). Certain thermophilic bacteria which did not possess LuxS were having Pfs e.g. *Fervidobacterium islandicum, Fervidobacterium nodosum, Thermosipho petrophila, Thermosipho melanesiensis* and *Thermotoga sp..* Maximum identity was observed with *Anoxybacillus spp*. and *Geobacillus spp*. (53–56%) while the identity was least with *Meiothermus ruber* (29%) and *Thermotoga spp*. (30–32%) which indicates that Pfs of *Anoxybacillus spp*. and *Geobacillus spp*. is more identical to that of mesophiles. Multiple sequence analysis showed no conservation among Pfs of all bacteria (Additional files 7 and 8).

**Table 2:** Exploring the presence of LuxS, Pfs, LuxP, LsrB, RbsB and SAH hydrolase in thermophilic eubacteria by using the protein sequences of LuxS, Pfs, LsrB of *E. coli* str. K-12 substr. MG1655, LuxP of *V. harveyi,* RbsB of *A. actinomycetemcomitans* SAH hydrolase of *Ralstonia solanacearum* as queries

Phylum	Bacteria	LuxS	Pfs	LuxP	LsrB	RbsB	SAH hydrolase	Possible role
		Identity (%)						
Aquificae	*Desulfurobacterium thermolithotrophum*	–	–	–	–	–	43	Metabolism (Detoxification of SAH)
	*Desulfurobacterium atlanticum*	–	–	–	–	–	–	Could not be determined
	*Desulfurobacterium pacificum*	–	–	–	–	–	–	Could not be determined
	*Sulfurihydrogenibium yellowstonense*	–	–	–	–	–	43	Metabolism (Detoxification of SAH)
	*Sulfurihydrogenibium azorense*	–	–	–	–	–	42	Metabolism (Detoxification of SAH)
	*Sulfurihydrogenibium subterraneum*	–	–	–	–	–	42	Metabolism (Detoxification of SAH)
	*Thermovibrio ammonificans*	–	–	–	–	–	42	Metabolism (Detoxification of SAH)
	*Thermovibrio ruber*	–	–	–	–	–	–	Could not be determined (Unknown)
Bacteroides	*Anaerophaga thermohalophila*	–	–	–	–	24	63	D-ribose transporter and metabolism (Detoxification of SAH)
Chloroflexi	*Chloroflexus aurantiacus*	–	–	–	–	–	–	Could not be determined
	*Chloroflexus aggregans*	–	–	–	–	52	43	D-ribose transporter and metabolism (Detoxification of SAH)
	*Thermomicrobium roseum*	–	–	–	–	–	43	Metabolism (Detoxification of SAH)
Deferribacteres	*Deferribacter thermophilus*	–	–	–	–	–	–	Could not be determined
	*Deferribacter desulfuricans*	–	–	–	–	–	40	Metabolism (Detoxification of SAH)
	*Deferribacter autotrophicus*	–	–	–	–	–	–	Could not be determined
Deinococcus-Thermus	*Marinithermus hydrothermalis*	42	–	–	–	–	42	AI-2 synthesis and metabolism (Detoxification of SAH)
	*Meiothermus ruber*	42	29	–	–	31	–	AI-2 synthesis
	*Meiothermus silvanus*	43	36	–	39	33	43	AI-2 synthesis and metabolism
	*Meiothermus chliarophilus*	44	33	–	–	32	41	AI-2 synthesis and metabolism
	*Meiothermus taiwanesis*	42	–	–	–	–	–	AI-2 synthesis
	*Meiothermus cerbereus*	41	–	–	–	–	–	AI-2 synthesis
	*Meiothermus rufus*	39	–	–	–	–	–	AI-2 synthesis
	*Oceanithermus profundus*	42	–	–	62	39	–	AI-2 synthesis
	*Thermus filiformis*	42	31	–	–	–	–	AI-2 synthesis
	*Thermus parvatiensis*	40	32	–	–	–	–	AI-2 synthesis
	*Thermus caliditerrae*	40	34	–	–	–	–	AI-2 synthesis
	*Thermus oshimai*	42	31	–	–	–	–	AI-2 synthesis
	*Thermus igniterrae*	41	33	–	–	–	–	AI-2 synthesis
	*Thermus scotoductus*	41	32	–	–	–	–	AI-2 synthesis
	*Thermus thermophilus*	39	32	–	–	–	–	AI-2 synthesis
	*Thermus amyloliquefaciens*	40	35	–	–	–	–	AI-2 synthesis
	*Thermus islandicus*	40	34	–	–	49	–	AI-2 synthesis
	*Thermus aquaticus*	40	–	–	–	–	–	AI-2 synthesis
Dictyoglomi	*Dictyoglomus thermophilum*	–	–	–	–	–	36	Metabolism (Detoxification of SAH)
Firmicutes	*Ammonifex thiophilus*	–	–	–	–	–	–	Could not be determined
	*Ammonifex degensii*	–	–	–	–	–	36	Metabolism (Detoxification of SAH)
	*Anoxybacillus geothermalis*	39	55	–	30	58	–	AI-2 synthesis
	*Anoxybacillus thermarum*	36	56	–	–	–	–	AI-2 synthesis
	*Anoxybacilllus amylolyticus*	39	56	–	–	59	–	AI-2 synthesis
	*Anoxybacillus suryakundensis*	36	55	–	–	–	–	AI-2 synthesis
	*Anoxybacillus flavithermus*	37	56	–	–	59	–	AI-2 synthesis
	*Caldanaerobacter subterraneus*	–	–	–	25	59	–	Sugar ABC transporter
	*Caldanaerobacter uzonensis*	–	–	–	–	–	–	Could not be determined
	*Caldicellulosiruptor saccharolyticus*	–	–	–	–	–	39	Metabolism (Detoxification of SAH)
	*Caldicellulosiruptor kronotskyensis*	–	–	–	–	–	39	Metabolism (Detoxification of SAH)
	*Caldicellulosiruptor bescii*	–	–	–	–	–	38	Metabolism (Detoxification of SAH)
	*Carboxydothermus hydrogenoformans*	–	–	–	–	–	39	Metabolism (Detoxification of SAH)
	*Carboxydothermus ferrireducens*	–	–	–	–	–	39	Metabolism (Detoxification of SAH)
	*Coprothermobacter proteolyticus*	–	–	–	–	61	37	D-ribose transporter and Metabolism (Detoxification of SAH)
	*Coprothermobacter platensis*	–	–	–	–	–	39	Metabolism (Detoxification of SAH)
	*Geobacillus caldoxylosilyticus*	40	54	–	–	–	–	AI-2 synthesis
	*Geobacillus stearothermophilus*	39	53	–	–	58	–	AI-2 synthesis
	*Geobacillus thermoglucosidasius*	38	53	–	–	57	–	AI-2 synthesis
	*Geobacillus kaustophilus*	39	55	–	–	58	–	AI-2 synthesis
	*Geobacillus subterraneus*	38	54	–	–	–	–	AI-2 synthesis
	*Geobacillus icigianus*	39	55	–	–	57	–	AI-2 synthesis
	*Geobacillus thermoleovorans*	39	55	–	–	58	–	AI-2 synthesis
	*Geobacillus thermonitrificans*	38	55	–	–	57	–	AI-2 synthesis
	*Halothermothrix orenii*	–	–	–	–	37	–	Metabolism (Detoxification of SAH)
	*Moorella thermoacetica*	–	–	–	29	29	36	Sugar ABC transporter and Metabolism (Detoxification of SAH)
	*Natranaerobius thermophilus*	–	–	–	–	59	39	D-ribose transporter and Metabolism (Detoxification of SAH)
	*Pelotomaculum thermopropionicum*	–	–	–	–	–	39	Metabolism (Detoxification of SAH)
	*Thermosediminibacter oceani*	39	37	–	–	29	–	AI-2 synthesis
	*Thermoanaerobacter kivui*	40	43	–	–	62	–	AI-2 synthesis
	*Thermoanaerobacter thermocopriae*	42	41	–	–	61	–	AI-2 synthesis
	*Thermoanaerobacterium thermosaccharolyticum*	42	43	–	–	55	–	AI-2 synthesis
	*Thermoanaerobacterium saccharolyticum*	43	47	–	–	–	–	AI-2 synthesis
	*Thermoanaerobacterium aotearoense*	43	46	–	–	–	–	AI-2 synthesis
	*Thermoanaerobacterium xylanolyticum*	43	46	–	–	–	–	AI-2 synthesis
	*Thermosinus carboxydivorans*	–	–	–	–	–	38	Metabolism (Detoxification of SAH)
Nitrospirae	*Thermodesulfovibrio aggregans*	–	–	–	–	–	41	Metabolism (Detoxification of SAH)
	*Thermodesulfovibrio thiophilus*	–	–	–	–	–	41	Metabolism (Detoxification of SAH)
	*Thermodesulfovibrio hydrogeniphilus*	–	–	–	–	–	–	Could not be determined
Proteobacteria	*Caminibacter mediatlanticus*	55	36	–	–	–	–	AI-2 synthesis
	*Caminibacter profundus*	–	–	–	–	–	–	Could not be determined
	*Caminibacter hydrogeniphilus*	–	–	–	–	–	–	Could not be determined
	*Desulfacinum hydrothermale*	–	–	–	–	–	–	Could not be determined
	*Desulfacinum infernurn*	–	–	–	–	–	–	Could not be determined
	*Hippea maritime*	–	–	–	–	–	42	Metabolism (Detoxification of SAH)
	*Hippea jasoniae*	–	–	–	–	–	41	Metabolism (Detoxification of SAH)
	*Hippea alviniae*	–	–	–	–	–	42	Metabolism (Detoxification of SAH)
	*Nitratiruptor* SB155-S	66	34	–	–	–	–	AI-2 synthesis
	*Thermochromatium tepidum*	–	–	–	–	–	–	Could not be determined
Thermodesulfobacteria	*Fervidobacterium islandicum*	–	27	–	–	51	37	D-ribose transporter and metabolism
	*Fervidobacterium gondwanense*	–	–	–	–	–	–	Could not be determined
	*Fervidobacterium changbaicum*	–	–	–	–	–	–	Could not be determined
	*Fervidobacterium nodosum*	–	27	–	–	–	37	Metabolism (Detoxification of SAH)
	*Kosmotoga pacifica*	–	–	–	–	53	–	D-ribose transporter
	*Kosmotoga olearia*	–	–	–	–	53	–	D-ribose transporter
	*Kosmotoga arenicorallina*	–	–	–	–	49	–	D-ribose transporter
	*Marinitoga piezophila*	–	–	–	–	–	36	Metabolism (Detoxification of SAH)
	*Marinitoga camini*	–	–	–	–	–	–	Could not be determined
	*Marinitoga hydrogenitolerans*	–	–	–	–	–	–	Could not be determined
	*Petrotoga mobilis*	–	–	–	–	53	36	D-ribose transporter and metabolism (Detoxification of SAH)
	*Petrotoga mexicana*	–	–	–	–	–	–	Could not be determined
	*Petrotoga halophila*	–	–	–	–	–	–	Could not be determined
	*Thermococcoides sp.*	–	–	–	–	–	–	Could not be determined
	*Thermodesulfatator indicus*	–	–	–	–	–	40	Metabolism (Detoxification of SAH)
	*Thermodesulfatator atlanticus*	–	–	–	–	–	41	Metabolism (Detoxification of SAH)
	*Thermodesulfatator autotrophicus*	–	–	–	–	–	40	Metabolism (Detoxification of SAH)
	*Thermosipho africanus*	–	–	–	–	50	38	D-ribose transporter and metabolism (Detoxification of SAH)
	*Thermosipho melanesiensis*	–	28	–	–	–	38	Metabolism (Detoxification of SAH)
	*Thermotoga petrophila*	–	32	–	–	49	–	D-ribose transporter and metabolism
	*Thermotoga maritime*	–	31	–	–	26	37	D-ribose transporter and metabolism (Detoxification of SAH)
	*Thermotoga neopolitana*	–	30	–	–	36	38	D-ribose transporter and metabolism (Detoxification of SAH)
	*Thermotoga naphthophila*	–	32	–	33	48	38	Sugar ABC transporter and metabolism (Detoxification of SAH)

Protein sequences with the names LuxS, S-ribosylhomocysteine lyase, S-ribosylhomocysteinase, MTA/SAH nucleosidase, Pfs, Autoinducer-2 binding protein, RbsB and D-ribose transporter were only considered for analysis

‘-‘indicates not present or less than 25% identical, however some sequences are mentioned in the table having very less identity (< 24%), as they are showing the similar names in NCBI database as the proteins of autoinducer-2 pathway

*M. ruber, M. silvanus, M. chilarophilus, O. profundus, T. islandicus, A. geothermalis, A. amylolyticus, A. flavithermus, G. stearothermophilus, G. thermoglucosidasius, G. kaustophilus, G. icigianus, G. thermoleovorans, G. thermonitrificans, T. oceani, T. kivui, T. thermocopriae, T. thermosaccharalyticum* have been found to have complete autoinducer-2 quorum sensing genes

*M. hydrothermalis, M. taiwanesis, M.cerbereus, M. rufus and T. aquaticus* have only LuxS protein and no other autoinducer-2 quorum sensing protein

*T. oshimai, T. filiformis, T. parvatiensis, T. calditerrae, T. igniterrae, T. scotoductus, T. thermophilus, T. amyloliquefaciens, T. islandicus* and *Anoxybacillus thermarum, A. suryakundensis, G. caldoxylosilyticus, T. saccharolyticum, T. aotearoense, T. xylanolyticum, C. mediatlanticus and Nitratiruptor sp.*possess both LuxS and Pfs protein and no other protein

*A. thermohalophila, C. aggregans, C. proteolyticus*, *N. thermophilus*, *K. pacifica, K. olearia*, *K. arenicorallina*, *P. mobilis, T. africanus, T. petrophila, T. maritima* and *T. neopolitana* have only RbsB protein while *C. subterraneus, M. thermoacetica* and *T. naphthophila* have both LsrB and RbsB protein but no LuxS

Phylum Aquificae, Deferribacteres, Dictyoglomi, Nitrospirae have none of the quorum sensing protein

**Fig. 4:**
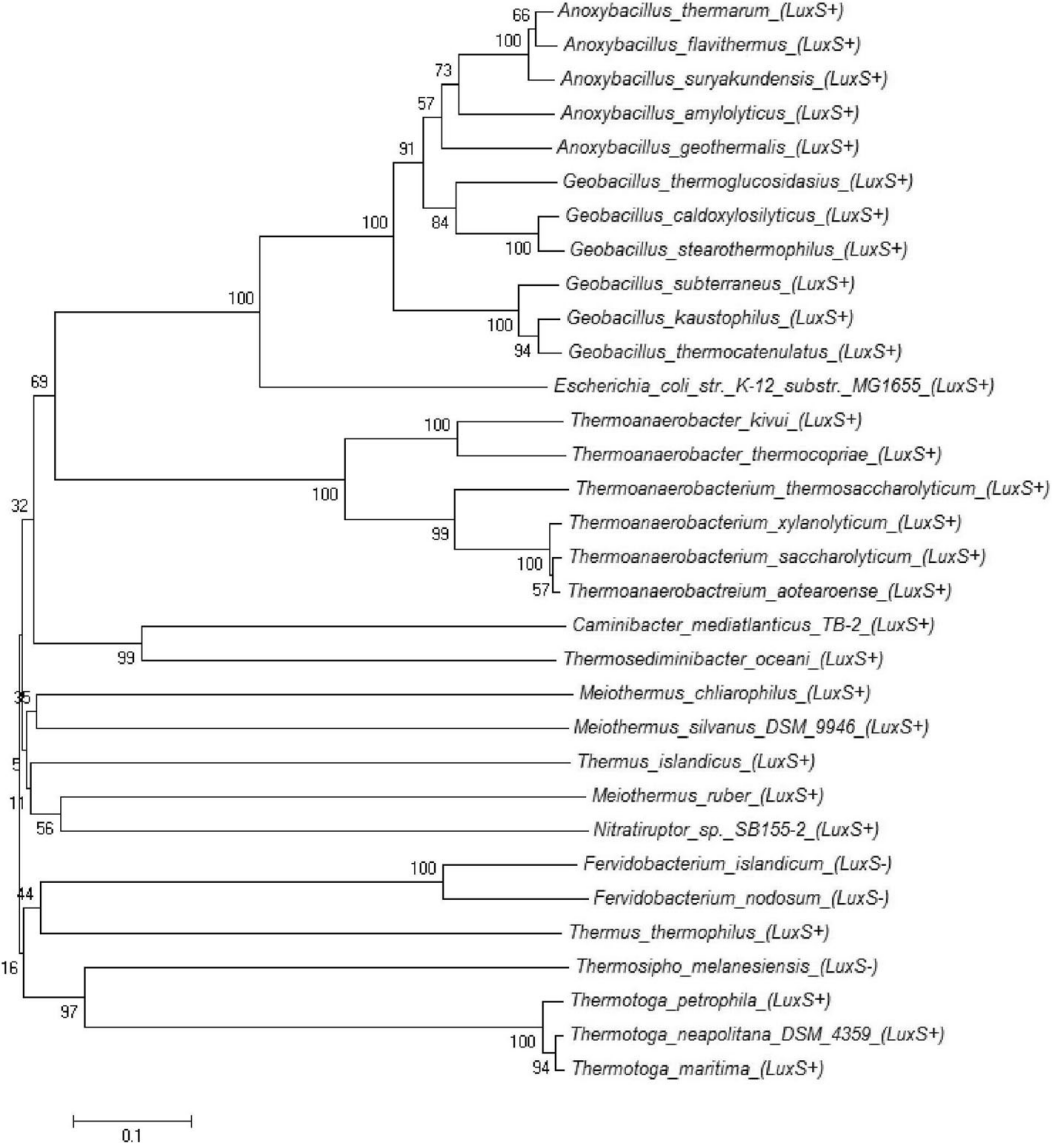
Evolutionary relationship of Pfs of thermophilic and *E. coli* str. K-12 substr. MG1655

Phylogenetic analysis to find the evolutionary relationship of MTA/SAH nucleosidase in thermophiles and *E. coli* str. K-12 substr. showed that among the LuxS positive species only Gram positive thermophiles, and *Thermotoga spp*. showed evolutionary relationship while all others were far distant in evolution (Fig. 4).

### Prevalence of SAH hydrolase

SAH hydrolase (S-adenosyl-L-homocysteine hydrolase) is involved in one step pathway for the conversion of SAH to homocysteine and adenosine without producing autoinducer-2 (Fig. 1). SAH hydrolase was found to be highly conserved among bacteria and was present in a large number of thermophiles (Tables1 and 2). Among archaea, SAH hydrolase was found in *Thermococcus barophilus*, *T. chitonophagus*, *T. gammatolerans*, *T. kodakarensis*, *T. litoralis*, *Pyrolobus fumarii*, *P. abyssi*, *P. furiosus*, *Pyrococcus horikoshii*, *Picrophilus torridus*, *Metallosphaera sedula and Aeropyrum pernix* with identity between 40 to 43% by using SAH hydrolase of *Ralstonia solanacearum* as query (Table 1). However none of the known quorum sensing system was found in thermophilic archaea. *M. silvanus and M. chliarophilus* had autoinducer-2 system as well as SAH hydrolase. All other thermophilic bacteria had only SAH hydrolase and no LuxS or autoinducer-2 system in them which shows that they have role in detoxification of SAH only (Table 2). MultAlin showed the highly conserved regions in SAH hydrolases (Additional files 9 and 10) of mesophilic and thermophilic bacteria.

**Fig. 5:**
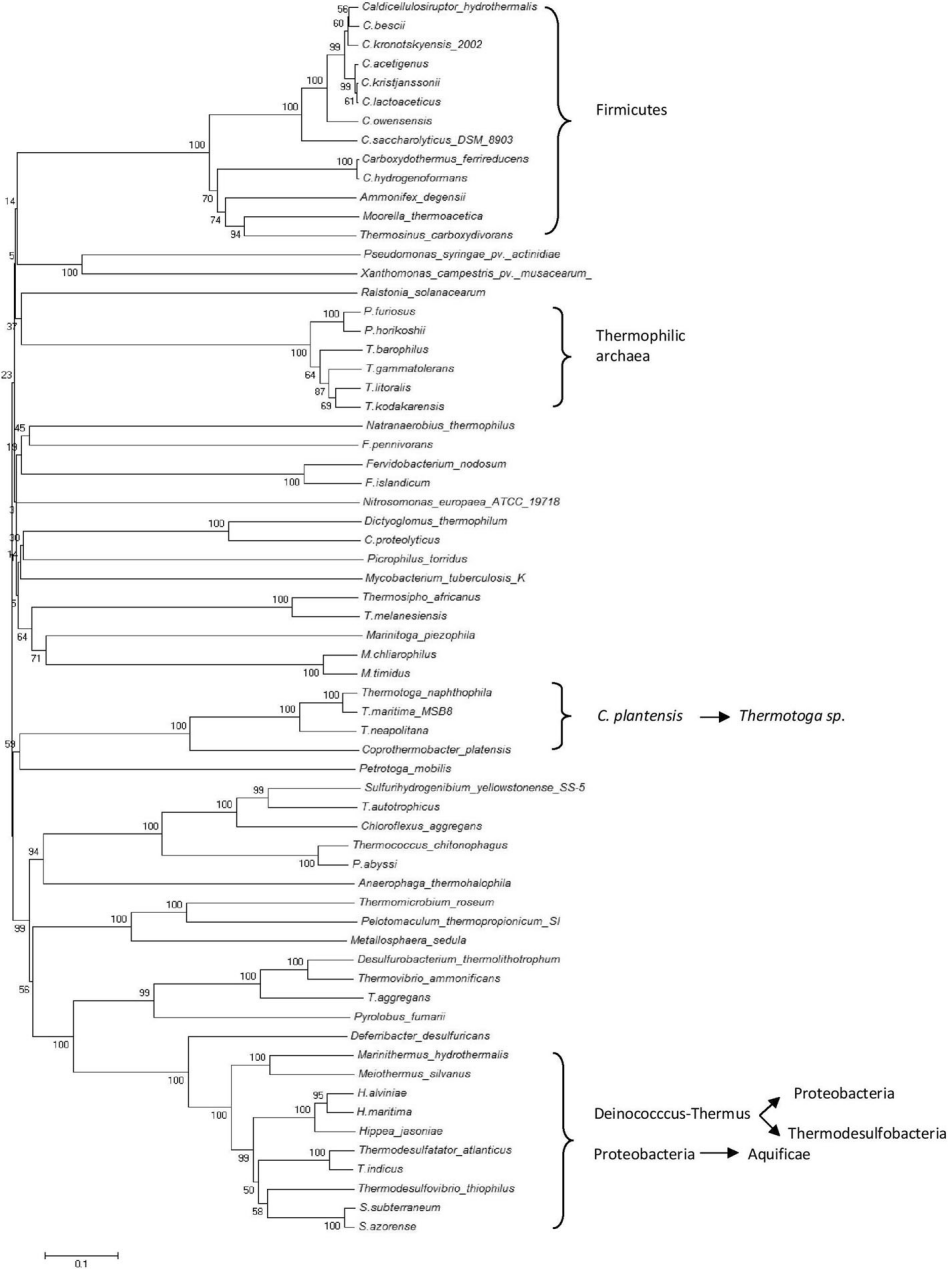
Evolutionary relationship of SAH hydrolases of thermophilic and mesophilc bacteria and archaea

Phylogenetic analysis showed four different clades (Fig. 5). LuxS negative *Firmicutes* formed a separate clade that indicates they all utilize one step pathway for the detoxification of SAH and their SAH hydrolases are evolutionarily identical. *Thermococcus spp*. and *Pyrococcus spp.* are forming a clade together that shows the high similarity and evolutionary linkage among their SAH hydrolases. Moreover microorganisms growing in similar environments may have acquired SAH hydrolase by horizontal gene transfer. A clade was formed by *Thermotoga spp.* sharing common ancestor (*C. platensis*). Last clade indicates events of horizontal gene transfer from *M. hydrothermalis* and *M. silvalnus* to Proteobacteria and Thermodesulfobacter and from Proteobacteria to Aquificae. *T. thiophilus*, *T. atlanticus* and *T. indicus* are sulfur reducing whereas *S. azorense* and *S. subterraneum* are sulfur oxidizing bacteria. They may have evolved together in evolution and may have acquired SAH hydrolase by horizontal gene transfer.

### Prevalence of AI-2 receptor proteins

#### LuxP and LsrB

**Fig. 6:**
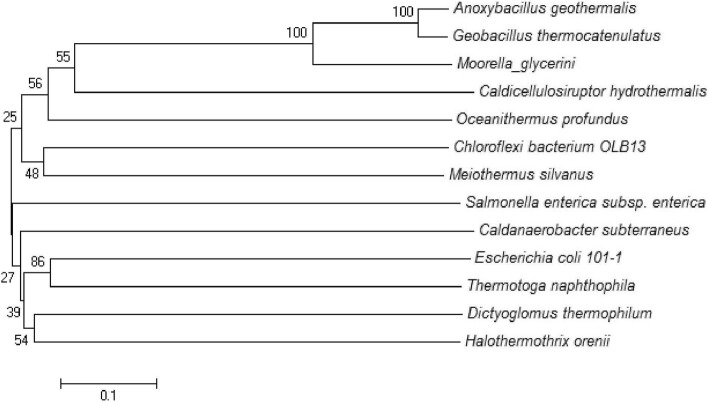
Evolutionary relationship of LsrB of thermophilic and mesophilc bacteria

LuxP was not found in any of the thermophilic bacteria through BLASTp using LuxP of *Vibrio harveyi* as query sequence. LsrB type autoinducer-2 ABC transporter substrate-binding proteins were present in *O. profundus, M. silvanus, A. geothermalis, T. napthophila, M. thermoacetica* and *C. subterraneus* and were absent in all other thermophilc bacteria. In LsrB positive strains the identity was 25–62% with query sequence. Maximum identity was with *O. profundus* (62%). LsrB type protein was present in few such thermophiles which did not possess LuxS e.g. *T. naphthophila, Moorella sp.* and *C. subterraneus* which indicates that these thermophiles can respond to autoinducer-2 but do not produce it or they are involved in pentose sugar transport. *M. silvanus* and *A. geothermalis* were the two thermophiles which possessed LuxS, Pfs and LsrB, hence the complete autoinducer-2 producing cascade. Further *Oceanithermus profundus* possessed LuxS and LsrB but no Pfs. All the LsrB sequences from mesophilic and thermophilic bacteria were aligned to find the conservation (Additional file 11). Few residues were conserved such as Lys-39, Gly-52, Gly-61, Pro-75, Gln-84, Ala-115, Gly-119 and Gly-262. Our results showed that the LsrB of *E. coli* str. K-12 substr. MG1655 had very less identity with thermophilic bacterial autoinducer-2 ABC substrate binding protein although the query cover was more than 90%. Phylogenetic analysis was performed to find the evolutionary relationship among autoinducer-2 binding LsrB protein in bacteria but no relationship was found except for *Firmicutes A. geothermalis*, *G. thermocatenulatus* that shared a common ancestor *M. glycerini* (Fig. 6).

### Rbs B

**Fig. 7:**
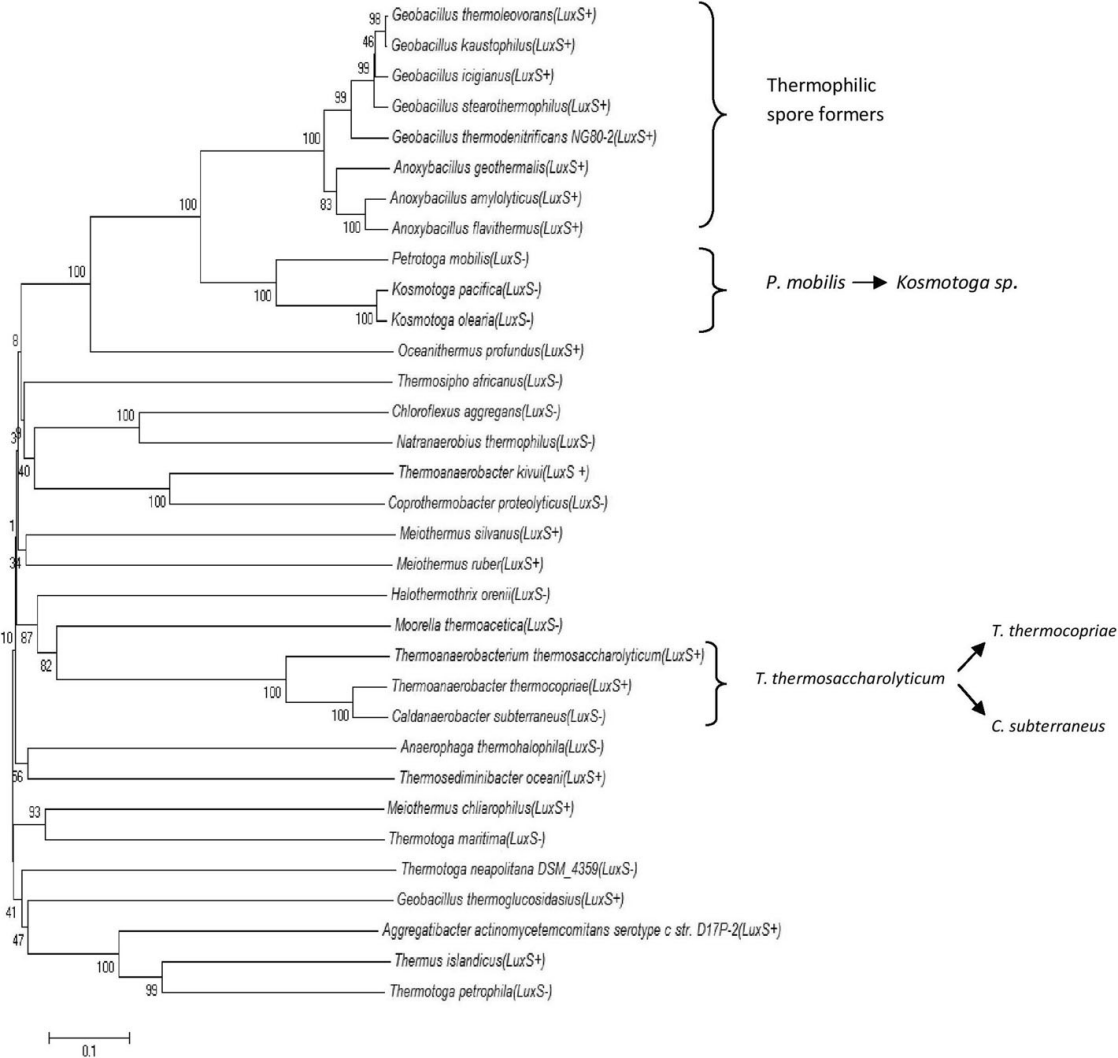
Evolutionary relationship of RbsB of thermophilic and mesophilc bacteria

In vitro competition assays demonstrated that LsrB and RbsB both are capable of binding AI-2 in *A. actinomycetemcomitans* [35]. RbsB homologs were searched in thermophilic bacteria by BLASTp using RbsB of *A. actinomycetemcomitans* as query sequence. High conservation was observed by MultAlin alignment (Additional file 12), maximum identity being with *Thermoanaerobacter spp*. (61–62%) and minimum with *Meiothermus spp.* (31–33%). This suggests that RbsB is highly conserved in bacteria, whether thermophiles or mesophiles. Phylogenetic analysis was done further to observe the evolutionary relationship (Fig. 7). Thermophilic spore formers are forming clade together. *Kosmotoga* spp. showed common ancestor *Petrotoga mobilis* while *T. thermocopriae* and *C. subterraneus* are sharing *T. thermosaccharolyticum* as ancestor. All of them belong to phylum *Firmicutes* which may indicate that *Firmicutes* have acquired RbsB by horizontal gene transfer, all other thermophiles were far apart in the evolution of LsrB*.*

### STRING analysis of LuxS

*Deinococcus-Thermus, Firmicutes* and *Proteobacteria* were the only phyla producing LuxS. Therefore, we have selected one member from each of these phyla for STRING analysis to find the proteins linked to LuxS as well as to find the possible role of LuxS in thermphilic bacteria. It is known that if Pfs and LuxS both are present than the pathway will lead to the production of DPD, a precursor of autoinducer-2. Sequence of LuxS protein of *M. ruber*, member of *Deinococcus-Thermus* phylum; *G. thermoglucosidasius,* member of *Firmicutes* phylum and *C. mediatlanticus,* member of *Proteobacteria* phylum were used for STRING analysis (Additional files 13, 14 and 15). Methionine synthase, cysteine synthase and MTA nucleosidase were linked to LuxS in all these thermophilic bacteria while cobalamin-independent methionine synthase, metE (5-methyltetrahydropteroyltri-L-glutamate) and homoserine dehydrogenase were linked to LuxS in *M. ruber* and *C. mediatlanticus* only. O-acetyl homoserine sulfhydrylase and cys/met metabolism pyridoxial phosphate dependent protein were present in *M. ruber* and *G. thermoglucosidasius* only. Presence of both LuxS and S-adenosylhomocysteine nucleosidase in all of them indicates that they are using two step pathways for the detoxification of SAH (Fig. 1). Presence of LuxS may further leads to the production of AI-2 in these thermophilic bacteria.

## Discussion

The way thermophilic bacteria adapt themselves in hot environment has become the topic of interest since 1960’s after the discovery of *T. aquaticus*. Presence of evidences for quorum sensing in mesophiles demands the investigation of quorum sensing mechanism(s) utilized by thermophiles. Thermophilic bacteria viz. *T. thermophilus* and thermophilic archaea viz. *P. furiosus* have been shown to form biofilms [45]. These thermophiles with other thermophilic biofilm formers are included in this study to suggest that these may interact with each other by quorum sensing. Previous work on the presence of peptide based quorum sensing in *T. maritima* [7], temperature dependent formation of AI-2 in *T. maritima* and archaean *P. furiosus* [11] and the expression of LuxS in *C. mediatlanticus* led us to search for quorum sensing systems present in thermophilic community.

In this study, the most prevalent quorum sensing systems were investigated. AI-1 system was not found in thermophiles which is in accordance with the results of Schopf et al. [10] that suggests the heat labile nature of AI-1 system. However, few thermophilic eubacteria showed traces for the presence of peptide based quorum sensing system (Table 2) but the identity with their mesophilic counterparts was very low. Only few members of phyla *Firmicutes, Deinococcus-Thermus, Chloroflexi* and *Thermodesulfobacteria* showed some identity with the response regulator AgrA and ComA but none of them showed any good identity with receptor histidine kinases which may suggest the presence of incomplete two component system in thermophilic eubacteria or they do not posseses any peptide based system or there may be some unknown proteins involved in two component peptide based system for thermophiles.

Search for MTA/SAH nucleosidase (Pfs), autoinducer-2 synthase (LuxS) and SAH hydrolase in the proteomes of thermophilic bacteria showed that the universal autoinducer-2 type of quorum sensing is the mode of communication. Pfs and SAH hydrolase are important enzymes like LuxS as they play role in the detoxification of SAH [20–22]. The *Pfs* mutant of *Neisseria meningitidis* completely stopped the release of AI-2 due to the accumulation of inhibitors such as MTA and toxic SAH [23]. These results were further supported by the previous work in which Pfs and LuxS inhibitors showed reduction in biofilm formation and did not produce autoinducer-2 [24–26]. Absence of LuxS and presence of Pfs and SAH hydrolase in *Fervidobacterium islandicum, F. nodosum, Thermosipho melanesiensis, Thermotoga sp.* and an archean *Aeropyrum pernix* in this work predicts that Pfs and SAH hydrolase play role in the detoxification of SAH and they do not produce AI-2*.* Therefore, in these bacteria due to the lack of LuxS no autoinducer-2 is synthesized.

Phylogenetic analysis of LuxS in thermophiles and mesophiles did not correlate with their 16S rRNA phylogeny, that indicates LuxS is more conserved in bacteria of different phyla and environment and mesophilic bacteria may have acquired LuxS from thermophiles (Fig. 2). Further LuxS from themophilic bacteria of same phylum were evolutionarily closer than from other phyla. This finding was supported by the Perez-Rodriguez et al. [12] who showed the LuxS gene flow from thermophiles to mesophilic epsilonproteobacteria. This work showed the events of horizontal *luxS* gene transfer, as certain thermophilic bacteria inhabiting similar environment were having LuxS that was evolutionarily more identical. *Anoxybacillus spp*. and *Geobacilllus spp* prevalent in dairy industry, *B. anthracis* and *B. cereus* bearing close phenotypic and genotypic resemblance, *T. thermocopriae* and *C. perfringens* found in decaying vegetables, *M. hydrothermalis* and *O. profundus*, inhabitants of deep sea hydrothermal vent, *S. marcescens* and *E.coli*, pathogenic bacteria, *S. enterica* (human pathogen) and *P. rhabdus* (insect pathogen) were having more identical LuxS. Further, *V. harveyi*, *S. marcescens* and *E.coli* were nested within thermophilic lineage as *Nitratiruptor sp.* SB155–2 is known to be phylogenetically related to epsilon-proteobacterial pathogens [27]. Thus this evolutionarily identical LuxS among proteobacteria may be the result of this relation.

Further, this study showed that some thermophilic bacteria viz. *M. hydrothermalis*, *M. taiwanesis, M. cerbereus, M. rufus, O. profundus* and *T. aquaticus* have LuxS but no Pfs. However, except *M. hydrothermalis* all of them possess SAH hydrolase*.* This indicates that here the detoxification of S-adenosylhomocysteine (SAH) occurs through SAH hydrolase since Pfs is absent in them. However, except *O. profundus* all of them lack the known receptors for AI-2 which predicts that they may not be involved in AI-2 type quorum sensing and their LuxS carries out the metabolic process such as the production of homocysteine for converting it back to methionine in AMC. However, the possibility of the presence of some unknown AI-2 receptors in them cannot be ruled out.

Multiple sequence alignment of LuxS of thermophilic and mesophilic bacteria showed the presence of invariant HXEEH motif which is in accordance with the work done by Keersmaecker et al. [28]. Further, our analysis showed that all thermophilic bacteria possess amino acids in the similar positions as in persistant hub which is the characterstic of extremophilic LuxS protein as described by Bhattacharyya and Vishveshwara [29]. *M. silvanus* and *M. chilarophilus* both showed autoinducer-2 synthesizing complete pathway as well as SAH hydrolases. Presence of both pathways in these two thermophiles allows them to recycle methionine more economically which is further supported by the work of Schell et al. [30] that showed the presence of both of these pathways in *Bifidobacterium longum*. Except *M. silvanus* and *M. chilarophilus,* all other thermophiles lacking complete autoinducer-2 system have SAH hydrolases which indicates that they may have role in detoxification of SAH to homocysteine and adenosine without synthesizing AI-2. Our results are further supported by Winzer et al. [31] who showed the presence of only SAH hydrolase and no autoinducer-2 synthesizing machinery in *Leptospira interrogans.* Nevertheless, the possibility of unknown mechanisms to synthesize AI-2 exists.

LuxP was not found in any of the thermophilic bacteria, corroborating the previous work showing the LuxP type receptors are only limited to Vibrionales and Oceanospirillales [32, 33].

Another well known receptor for autoinducer-2 was found to be LsrB. There are evidences for the presence of LsrB in *Enterobacteriaceae* and *Rhizobiaceae* and *Bacillaceae* families [34]. Autoinducer-2 ABC transporter which shares identity with LsrB ABC transporter was found in some thermophilic bacteria in this study. *M. silvanus, O. profundus, A. geothermalis, C. subterraneus, M. thermoacetica* and *T. naphthophila* were the only thermophiles having autoinducer-2 ABC transporter identical to LsrB. Our results showed that *M. silvanus*, *A. geothermalis* and *O. profundus* possess both Pfs and LuxS as well as LsrB, complete autoinducer-2 quorum sensing circuit which can produce as well as detect autoinducer-2. However, all of these thermophiles posseses RbsB as well. It indicates that they may utilize two receptors for autoinducer-2 detection like in *Aggegatibacter actinomycetemcomitans* [35] where LsrB protein competes with the LuxP of *Vibrio harveyi* during autoinducer-2 bioassay and inhibits the interaction of AI-2 with LuxP. They further showed that LsrB competes with LuxP for the AI-2 produced by *V. harveyi* while RbsB competes with LuxP for the AI-2 produced by *A. actinomycetemcomitans.* It may be concluded from our work that *C. subterraneus, M. thermoacetica* and *T. naphthophila* possess autoinducer-2 ABC transporter protein which is identical to LsrB ABC transporter but they all lacked LuxS protein which indicates that they might respond to autoinducer-2 produced by other bacteria in biofilms without producing it themselves. This is in accordance with the results of Rezzonico and Duffy [32] which showed the presence of orphan *lsr* operon in *R. sphaeroides* and *S.meliloti* that lack *luxS.* It may be possible for bacteria to detect the AI-2 signal even if it does not produce it. *Pseudomonas aeruginosa* does not contain *luxS* but in the presence of enzymatically produced AI-2 or in co-culture with *Streptococcus mitis*, some of its virulence gene promoters were upregulated [36, 37]. Similar findings were observed in some mixed biofilm formation [38].

In previous reports, RbsB has been speculated as another receptor for autoinducer-2 in *Borellia burgdorferi* and *Actinobacillus actinomycetemcomitans* [35, 39]. Our study found a large number of thermophilic bacteria having D-ribose binding protein identical to RbsB of *A. actinomycetemcomitans* (Additional file 12). Ribose ABC-transporter is distributed ubiquitously among thermophilic bacteria regardless of the prevalence of Pfs and LuxS and these results are in accordance with the previous work [32]. It may thus work as the autoinducer-2 receptor in thermophilic bacteria lacking LuxP and LsrB.

Furthermore, there are certain mesophilic bacteria (*Actinobacillus pleuropneumoniae, Borrelia burgdorferi, Helicobacter pylori, Mycobacterium avium and Streptococcus spp.*) which are known to form biofilms and other quorum sensing phenotypes and produce AI-2 but lack the known LuxP, LsrB and RbsB like receptors [40–43]. Similarly in many thermophiles no known receptor for AI-2 has been found, so unknown alternative receptors might exist for them. Presence of LuxS and autoinducer-2 receptor in thermophiles may explain their role in quorum sensing. Presence of autoinducer-2 receptor but lack of autoinducer-2 synthase in certain thermophiles may explain their interactions with neighbours in biofilms regardless of producing autoinducer-2 synthase but responding to autoinducer-2. Peptide based or other unknown quorum sensing systems may be the mode of communication used by some thermophilic archaea as no autoinducer-1 or autoinducer-2 system is present in them.

## Conclusion

Thermophiles are one of the major groups of extremophile. They are known to form biofilms at high temperature. Identifying the phenomenon to form biofilms at high temperature may reveal how these microorganisms adapted themselves at such adverse conditions. As mentioned in previous studies, AI-1 and oligopeptides are damaged by hydrolytic cleavage whereas; AI-2 is quiet stable at different pH range and temperatures [12, 44]. Therefore AI-2 might be the mode of communication used by thermophilic bacteria. Other unknown quorum sensing systems may be prevalent in thermophiles which have to be explored further. To conclude, our study is an attempt to understand the mechanism of quorum sensing used by thermophiles as information regarding quorum sensing has not been explored in most of the thermophiles. Further this information can be utilized for developing strategies to disrupt the biofilms formed by these thermophiles at high temperature.

## Methods

### Analysis for the presence of quorum sensing systems (AI-1, peptide based, AI-2) in thermophiles

A total of 106 thermophilic bacteria from 10 different phyla and 21 thermophilic archaea from 3 different phyla were analyzed for the presence of quorum sensing proteins through NCBI protein database (http:// www.ncbi.nlm.nih.gov). Homologues of autoinducer synthases (LuxI for AI-1 and LuxS for AI-2), their receptors (LuxR for AI-1 and LuxP, LsrB and RbsB for AI-2), MTA/SAH nucleosidase (Pfs) and SAH hydrolase were searched in thermophiles through BLASTp (http://blast.ncbi.nlm.nih.gov/Blast.cgi). Quorum sensing signaling peptides were explored using Quorumpeps database [14]. Protein sequences of receptor histidine kinases and response regulators involved in peptide based quorum sensing systems viz. AgrC/AgrA, FsrC/FsrA, RapC/RapA, ComP/ComA and ComD/ ComE were used as queries for BLASTp.

Protein sequences of LuxI/LuxR of *Vibrio fischeri* (accession number YP_206882.1 and AAW87995.1), LuxS of *Escherichia coli* str. K-12 substr. MG1655 (accession number NP_417172.1), LuxP of *V. harveyi* (accession number AAA20837.2), LsrB of *E. coli* str. K-12 substr. MG1655 (accession number AMC98721.1), RbsB of *Aggregatibacter actinomycetemcomitans* (accession number WP_050846150.1), Pfs protein of *E. coli* str. K-12 substr. MG1655 (accession number NP_414701.1), S-adenosyl-L-homocysteine hydrolase of *Ralstonia solanacearum* (accession no. KFZ93620.1), AgrC/AgrA of *Staphylococcus aureus* (accession number ABX60402.2 and ALY21623.1), FsrC/FsrA of *Enterococcus faecalis* (accession number AFO44434.1 and AFO44436.1), RapC/RapA and ComP/ComA of *Bacillus subtilis* (accession number AAT75294.1, KIX82477.1, AKN15283.1 and BAB13494.1) and ComD/ ComE (accession number AAC44896.1 and APJ35576.1) of *Streptococcus pneumoniae* were used as queries for BLASTp.

Sequences of quorum sensing proteins in thermophiles having query cover more than 85% with identity more than 35% (except for Pfs and LsrB, as during BLASTp analysis some of the protein sequences in thermophiles are showing names similar to Pfs and LsrB but with identity less than 35%) were aligned using MultAlin with the LuxS homologes in mesophilic bacteria such as *Serratia marcescens* (ALE94556.1)*, Campylobacter jejuni* subsp. jejuni ATCC 33560 (EIB44085.1), *Salmonella enterica (*AAR88507.1*), E. coli* str. K-12 substr. MG1655 (NP_417172.1), *Bacillus cereus* H3081.97 (EDZ54884), *Bacillus anthracis* str. Ames (AAP28724.1), *Photorhabdus luminescens* (OCA55726.1)*, V. harveyi* (AAD17292.1). Further MultAlin was done by aligning SAH hydrolases of thermophiles and mesophiles (*Pseudomonas syringae* pv. actinidiae, accession no. GAO96569.1; *Xanthomonas campestris* pv. musacearum NCPPB 4384, accession no. KFA32367.1; *Nitrosomonas europaea* ATCC 19718, accession no. CAD84571.1; *Mycobacterium tuberculosis* K, accession no. AIB49973.1) having SAH hydrolase to find the conservation in all of them.

### Phylogenetic analysis

Phylogenetic trees were constructed for LuxS, Pfs, LsrB, RbsB, SAH hydrolase as well as for 16S rRNA genes of thermophiles and mesophiles by aligning the sequences using MEGA (Molecular Evolutionary Genetics Analysis) 6.0 software and neighbor-joining (NJ) method [15]. Bootstrap test using 1000 replicates was shown next to the branches [16]. The evolutionary distances were computed using the p-distance method [17].

### STRING analysis of LuxS

STRING (Search Tool for the Retrieval of Interacting Genes/Proteins) analysis [18, 19] of LuxS of one representative member of each phylum of thermophilic bacteria was performed to find the interacting protein. STRING analysis was used to find the common proteins linked to LuxS in different phylum.

## Additional files

Additional file 1: Peptide based quorum sensing systems. (PDF 10 kb)

Additional file 2: Prevalence of peptide based quorum sensing system in thermophilic eubacteria. (PDF 125 kb)

Additional file 3: Multiple sequence alignment of AgrA protein from Staphylococcus aureus and thermophilic eubacteria by MultAlin. (PDF 40 kb)

Additional file 4 Multiple sequence alignment of FsrA protein from Enterococcus faecalis D32 and thermophilic eubacteria by MultAlin. (PDF 30 kb)

Additional file 5: Multiple sequence alignment of ComA protein from Bacillus subtilis and thermophilic eubacteria by MultAlin. (PDF 120 kb)

Additional file 6: Multiple sequence alignment of LuxS protein from mesophilic and thermophilic eubacteria by MultAlin. Invariant residues are highlighted in red colour. The conserved HTLEH motif and other conserved residues are highlighted within boxes. (PDF 111 kb)

Additional file 7: Multiple sequence alignment of MTA/SAH nucleosidase from mesophilic and thermophilic eubacteria by MultAlin. (PDF 88 kb)

Additional file 8: Multiple sequence alignment of MTA/SAH nucleosidase from thermophilic eubacteria by MultAlin. There is no conservation among Pfs protein sequences. (PDF 138 kb)

Additional file 9: Multiple sequence alignment of SAH hydrolase from thermophilic and mesophilic eubacteria by MultAlin. High conservation among SAH hydrolases has been observed. (PDF 274 kb)

Additional file 10: Multiple sequence alignment of SAH hydrolase from thermophilic eubacteria by MultAlin. High conservation among SAH hydrolases has been observed. (PDF 323 kb)

Additional file 11: Multiple sequence alignment of LsrB from mesophilic and thermophilic eubacteria by MultAlin. (PDF 105 kb)

Additional file 12: Multiple sequence alignment of RbsB protein from mesophilic and thermophilic eubacteria by MultAlin. Conserved residues are highlighted in red within boxes. (PDF 142 kb)

Additional file 13: STRING analysis of LuxS protein of Meiothermus ruber. (PDF 202 kb)

Additional file 14: STRING analysis of LuxS protein of Geobacillus thermoglucosidasius. (PDF 225 kb)

Additional file 15 STRING analysis of LuxS protein of Caminibacter mediatlanticus. (PDF 90 kb)

Additional file 16: NCBI accession number of genomes of thermophililc archaea used in the study. (PDF 24 kb)

Additional file 17: NCBI accession number of genomes of thermophililc bacteria used in the study. (PDF 137 kb)

## Abbreviations

AI-1: Autoinducer-1
AI-2: Autoinducer-2
AMC: Activated methyl cycle
MTA: Methylthioadenosine
SAH hydrolase: S-adenosyl-L-homocysteine hydrolase
SAH nucleosidase: S-adenosylhomocysteine nucleosidase
DPD: 4,5-dihydroxy-2,3-pentanedione
NCBI: National Center for Biotechnology Information
BLAST: Basic Local Alignment Search Tool
MEGA: Molecular Evolutionary Genetics Analysis
STRING: Search Tool for the Retrieval of Interacting Genes/Proteins
AHL: Acyl homoserine lactone
ABC transporter: ATP-binding cassette transporter
metE: 5-methyltetrahydropteroyltri-L-glutamate
