# Supplementary material for: Quorum sensing in thermophiles: prevalence of autoinducer-2 system
Source: BMC Microbiol. 2018 Jun 28;18:62. doi: 10.1186/s12866-018-1204-x (PMC6022435; doi:10.1186/s12866-018-1204-x)
Supplement: Supplementary file 1 — Peptide based quorum sensing systems. (PDF 10 kb) [file 12866_2018_1204_MOESM1_ESM.pdf]

**Bacteria**	**Receptor histidine kinase**	**Response regulator**
Staphylococcus aureus	AgrC	AgrA
Enterococcus faecalis	FsrC	FsrA
Bacillus subtilis	RapC	RapA
Bacillus subtilis	ComP	ComA
Streptococcus pneumoniae	ComD	ComE
