# Supplementary material for: Quorum sensing in thermophiles: prevalence of autoinducer-2 system
Source: BMC Microbiol. 2018 Jun 28;18:62. doi: 10.1186/s12866-018-1204-x (PMC6022435; doi:10.1186/s12866-018-1204-x)
Supplement: Supplementary file 2 — Prevalence of peptide based quorum sensing system in thermophilic eubacteria. (PDF 125 kb) [file 12866_2018_1204_MOESM2_ESM.pdf]

AgrC receptor histidine kinase (*Staphylococcus aureus*)				
**Phylum**	**Bacteria**	**Function**	**Query cover**	**Identity**
Deinococcus-Thermus	*Meiothermus rufus*	Histidine kinase	45	23
	Meiothermus chliarophilus	Histidine kinase	44	24
	Oceanithermus profundus	Histidine kinase	16	31
Firmicutes	*Halothermothrix orenii*	Histidine kinase	31	26
	Thermoanaerobacterium thermosaccharolyticum	Histidine kinase	46	28
	Thermoanaerobacterium saccharolyticum	Histidine kinase	52	26
	Thermoanaerobacterium aotearoense	Histidine kinase	42	25
**AgrA response regulator (*Staphylococcus aureus*)**				
Thermodesulfobacteria	*Thermodesulfatator atlanticus*	DNA binding protein	33	25
	Kosmotoga pacifica	DNA binding protein	90	25
Firmicutes	*Thermoanaerobacterium xylanolyticum*	DNA binding protein	47	30
	Natranaerobius thermophilus	DNA binding response protein	94	25
	Pelotomaculum thermopropionicum	Response regulator	89	24
	Geobacillus kaustophilus	DNA binding response protein	89	27
	Geobacillus caldoxylosilyticus	DNA binding response regulator	90	26
	Carboxydothermus ferrireducens	Response regulator	38	28
	Carboxydothermus hydrogenoformans	Response regulator	30	28
	Caldanaerobacter subterraneus	Response regulator	48	28
	Anoxybacillus flavithermus	Response regulator	22	30
	Anoxybacillus thermarum	Response regulator	29	23
	Anoxybacillus suryakundensis	Response regulator	22	30
	Ammonifex degensii	DNA binding response regulator	80	30
Bacteroides	*Anaerophaga thermohalophila*	DNA binding response regulator	68	25

**FsrC receptor histidine kinase (*Enterococcus faecalis*)**				
Chloroflexi	*Chloroflexus sp.*	Two component sensor histidine kinase	14	32
**FsrA response regulator (*Enterococcus faecalis*)**				
Thermodesulfobacteria	*Marinitoga piezophila*	DNA binding response regulator	95	23
Nitrospirae	*Thermodesulfovibrio thiophilus*	DNA binding response regulator	50	21
Firmicutes	*Moorella thermoacetica*	DNA binding response regulator	69	25
	Halothermothrix orenii	DNA binding response regulator	15	34
	Geobacillus caldoxylosilyticus	LytR family transcriptional regulator	56	23
	Caldanaerobacter subterraneus	DNA binding response regulator	61	25
**RapC receptor histidine kinase (*Bacillus subtilis*)**				
Not present				
**RapA response regulator (*Bacillus subtilis*)**				
Not present				
**ComP receptor histidine kinase domain (*Bacillus subtilis*)**				
Firmicutes	*Anoxybacillus thermarum*	Peptide ABC transporter substrate binding protein	40	29
	Anoxybacillus suryakundensis	Peptide ABC transporter substrate binding protein	40	29
	Anoxybacillus flavithermus	Peptide ABC transporter substrate binding protein	40	31
**ComA response regulator (*Bacillus subtilis*)**				

Chloroflexi	*Chloroflexus aggregans*	DNA binding response regulator	98	32
	Thermomicrobium roseum	DNA binding response regulator	99	30
Deinococcus-Thermus	*Marinithermus hydrothermalis*	DNA binding response regulator	99	33
	Meiothermus ruber	DNA binding response regulator	99	33
	Meiothermus silvanus	DNA binding response regulator	99	32
	Meiothermus chilarophilus	DNA binding response regulator	99	32
	Meiothermus cerbereus	DNA binding response regulator	99	34
	Meiothermus rufus	DNA binding response regulator	99	33
	Oceanithermus profundus	Two component transcriptional regulator	99	33
	Thermus filiformis	DNA binding response regulator	99	33
	Thermus parvatiensis	DNA binding response regulator	38	29
	Thermus calditerrae	DNA binding response regulator	99	31
	Thermus oshimai	DNA binding response regulator	99	32
	Thermus igniterrae	DNA binding response regulator	99	32
	Thermus scotoductus	DNA binding response regulator	99	32
	Thermus thermophilus	DNA binding response regulator	99	33
	Thermus amyloliquefaciens	DNA binding response regulator	99	32
	Thermus islandicus	DNA binding response regulator	99	32
	Thermus aquaticus	DNA binding	99	33

		response regulator		
Firmicutes	*Anoxybacillus geothermalis*	DNA binding response regulator	100	29
	Anoxybacillus thermarum	DNA binding response regulator	98	28
	Anoxybacilllus amylolyticus	DNA binding response regulator	100	37
	Anoxybacillus suryakundensis	DNA binding response regulator	100	38
	Anoxybacillus flavithermus	DNA binding response regulator	99	33
	Caldanaerobacter subterraneus	DNA binding response regulator	96	30
	Caldicellulosiruptor saccharolyticus	DNA binding response regulator	93	30
	Caldicellulosiruptor kronotskyensis	DNA binding response regulator	49	27
	Caldicellulosiruptor bescii	DNA binding response regulator	93	31
	Carboxydothermus hydrogenoformans	DNA binding response regulator	39	26
	Carboxydothermus ferrireducens	DNA binding response regulator	94	25
	Geobacillus caldoxylosilyticus	DNA binding response regulator	100	38
	Geobacillus thermoglucosidasius	DNA binding response regulator	93	30
	Geobacillus kaustophilus	DNA binding response regulator	100	28
	Geobacillus icigianus	DNA binding response regulator	100	37
	Geobacillus thermoleovorans	DNA binding response regulator	100	27
	Geobacillus thermonitrificans	DNA binding response regulator	45	30
	Halothermothrix orenii	DNA binding response	97	30

		regulator		
	Moorella thermoacetica	Transcriptional regulatory protein DeqV	98	34
	Natranaerobius thermophilus	DNA binding response regulator	99	29
	Pelotomaculum thermopropionicum	Response regulator	96	32
	Thermosediminibacter oceani	Two component transcriptional regulator, AraC family	49	29
	Thermoanaerobacter thermocopriae	Two component transcriptional regulator, LuxR family	96	30
	Thermoanaerobacterium thermosaccharolyticum	DNA binding response regulator	96	31
	Thermoanaerobacterium saccharolyticum	DNA binding response regulator	96	31
	Thermoanaerobacterium aotearoense	DNA binding response regulator	96	31
	Thermoanaerobacterium xylanolyticum	DNA binding response regulator	96	38
	Thermosinus carboxydivorans	DNA binding response regulator	95	31
Nitrospirae	*Thermodesulfovibrio aggregans*	Fis family transcriptional regulator	48	22
Proteobacteria	*Desulfacinum infernurn*	Fis family transcriptional regulator	83	26
	Hippea jasoniae	Fis family transcriptional regulator	60	24
	Nitratiruptor SB155-S	DNA binding response regulator	28	39
Thermodesulfobacteria	*Thermodesulfatator indicus*	Response regulator	48	27
	Thermodesulfatator autotrophicus	Hybrid sensor histidine kinase/ response regulator	40	28
	Thermosipho africanus	DNA binding response regulator	32	27
	Thermosipho melanesiensis	Response regulator	55	29

	Thermotoga maritima	Two component system response regulator	40	29
	Thermotoga neopolitana	Response regulator	32	33
	Thermotoga naphthophila	Response regulator	32	33
**ComD receptor histidine kinase (*Streptococcus pneumoniae*)**				
Chloroflexi	*Chloroflexus aggregans*	Hybrid sensor histidine kinase/ response regulator	13	23
Firmicutes	*Ammonifex degensii*	Two component sensor histidine kinase	9	35
	Anoxybacillus geothermalis	Histidine kinase	32	28
	Anoxybacilllus amylolyticus	Histidine kinase	34	26
	Caldanaerobacter subterraneus	Histidine kinase	29	31
	Geobacillus stearothermophilus	Histidine kinase	32	28
	Halothermothrix orenii	Signal transduction histidine kinase	30	28
	Moorella thermoacetica	Signal transduction histidine kinase	55	24
	Thermoanaerobacterium saccharolyticum	Histidine kinase	27	26
	Thermoanaerobacterium aotearoense	Histidine kinase	27	26
	Thermosinus carboxydivorans	DNA binding response regulator	51	29
**ComE response regulator (*Streptococcus pneumoniae*)**				
Deinococcus-Thermus	*Meiothermus chliarophilus*	Response regulator	17	40
Firmicutes	*Anoxybacilllus amylolyticus*	DNA binding response regulator	76	25
	Anoxybacillus flavithermus	Two component system response regulator	26	23
	Halothermothrix orenii	LuxR family transcriptional regulator	47	22
	Thermosinus carboxydivorans	DNA binding response regulator	51	29
Thermodesulfobacteria	*Thermotoga petrophila*	Two component system response regulator	48	23
	Thermotoga maritima	DNA binding response regulator	34	22
