# Supplementary material for: Quorum sensing in thermophiles: prevalence of autoinducer-2 system
Source: BMC Microbiol. 2018 Jun 28;18:62. doi: 10.1186/s12866-018-1204-x (PMC6022435; doi:10.1186/s12866-018-1204-x)
Supplement: Supplementary file 3 — Multiple sequence alignment of AgrA protein from Staphylococcus aureus and thermophilic eubacteria by MultAlin. (PDF 40 kb) [file 12866_2018_1204_MOESM3_ESM.pdf]

```
                  1        10        20        30        40        50        60        70        80        90        100       110       120       130
                  |--------+---------+---------+---------+---------+---------+---------+---------+---------+---------+---------+---------+---------|
  Staphylococcus  MKIFICEDDPKQRENMVTIIKNYIMIEEKPMEIALATDNPYEVLEQAKNMNDIGCYFLDIQLSTD-INGIKLGSEIRKHDPVGNIIFVTSHSELTYLTFVYKVAAMDFIFKD-DPAELRTRIIDCLETAH
Parageobacillus  LKIVVADDDASSRILLRHFIRFL-----PQYKVVGEAISGEEFVRLVLEEKP-DIALVDIKMPD--LDGMEAVKICKQSLPALQVIFTTGYDE--FAVKAFEIAATDYIVKPIERTRLFHALEKARKLIE
     Geobacillus  LKIVIADDDASSRSILRHFIHLF-----PNYDVVAEATSGEEFLQLVLQEQP-DIVLVDINMPG--LDGMEAVKICRQLFPALQVIFITGYDE--FAVEAFEVSATDYIVKPIERTRLFYALEKARKLIE
       Kosmotoga  IKAIVVDDEIIARKAIIELIEDY-----QNIEVMGEASSAEEALDIIKTKKP-NVVFLDIQLPG--FNGVEFVELLNRLEEKVLVVFISAYDE--YALDAFEVDAVDYLMKPVSPERFAVTVERIEKV--
    Pelotomaculum  LKALIVDDEYPARQELRYALSGF-----GNVEIVGEATNAQEAMALIRALDY-QVLFLDISMPG--MNGLDLGAAIQELPRRPYIIFVTAYDE--YAVSAFEVNAVDYILKPVEPKRLKKALDKVIKLTQ
  Natranaerobius  MKLLILEDENFTRKYLKELVQRN-----KNIDEVYATGDVNEALEIVGSELP-QIGLIDIELPEQYINGLEASRTAQQINPEMEFIFVTAYSQ--YALKSFEVHPYDYFVKPVGEENLLESVNNIVERIK
       Consensus  .K..!.#D#...R..l...i.........n.e.v.ea....Eal.......p....flDI.$p....#G.e.........p....!Fvtayd#..%a..a%e!.a.D%i.Kpv.p.rl........k...

                  131     140       150       160       170       180       190       200       210       220       230       240       250       260
                  |--------+---------+---------+---------+---------+---------+---------+---------+---------+---------+---------+---------+---------|
  Staphylococcus  TRLQLLSKDN----------------------SVETIELKRGSNSYYVQYDDIMFFESSTKSHRLIAHLDNRQIEFYG-NLKELSQL--DDRFFRCHNSFVVNRHNIESIDSKERIVYFKNKEHCYASVR
Parageobacillus  LSRQ-DHLSIKT--------------------KNKRLGIKSHNSILFLPMEDILFVEK--ESRKTVIHTANERYETTE-PLNKMENKL-DDYFFKTHRSYIVNLKKIVKIEP
     Geobacillus  MAKQCAAVKAKQ--------------------PSKRLGIRSKNSIVLLPMEDILFVEK--ESRKTVIHTANERYETTE-TLNEIEREL-DNCFFKTHRSYIINLKKIVKI
       Kosmotoga  -------LAKEG--------------------VLEKVPVKEDNLLEMVDLEEIFYFES--MDKKVYLHTKEDELEVYRYNLSELESSL-PSFFARVHKSFIVNLKKVRRFSS
    Pelotomaculum  EGAPAAKVLITGQEGPAVRIGDANGQAPQSQIKIDRIPAEKQGKTILVTGSDIFYAFT--EKDYVYLKTYSDKL-FTRFTLKELEARLNPQVFFRTHRCYLVNLHKVREI
  Natranaerobius  ANL-----------------------------SYKKLILDEPGEVLQIPMHKILFLEKIKEQKKVSIYLEDDEVYQVSKTLNELEEML-DINFVRSHRSYIVNKHKIRKITAISQKFYEAYFEN
       Consensus  .......................................k.......v...dI.%.e...e..kv..ht..d..e.....L.ele..l.d..Ffr.Hrs%i!Nlhk!r.i....................

                  26265
                  |---|
  Staphylococcus  NVKKI
Parageobacillus
     Geobacillus
       Kosmotoga
    Pelotomaculum
  Natranaerobius
       Consensus  .....
```
