# Supplementary material for: Quorum sensing in thermophiles: prevalence of autoinducer-2 system
Source: BMC Microbiol. 2018 Jun 28;18:62. doi: 10.1186/s12866-018-1204-x (PMC6022435; doi:10.1186/s12866-018-1204-x)
Supplement: Supplementary file 4 — Multiple sequence alignment of FsrA protein from Enterococcus faecalis D32 and thermophilic eubacteria by MultAlin. (PDF 30 kb) [file 12866_2018_1204_MOESM4_ESM.pdf]

```
             1       10        20        30        40        50        60        70        80        90        100       110       120       130
             |--------+---------+---------+---------+---------+---------+---------+---------+---------+---------+---------+---------+---------|
Enterococcus MSEQMAIYILEDQIIQAKALEALVTNILHSRNIYNETIHLFSRSDELLQVAHQDAQLNIFFLDIQMNNHIQAGFEVAKEIRKTDSESLIVFISTHTELVLTSYKYMVSALQFIQKNVDFLDFQKEVETCV
  Marinitoga         IIDDEYYARESLKDLI----HEMSDFN-IVGCYESIEEFMKKSKNN-EIHVIFLDIELPK--MNGIKAAKYL----NQHKIVFVTAYSEYAVDAFE--VNALDYITKPVSEERF---IET-I
   Consensus ........Ii#D#iiaaeaLeaL!....Hernd%N.i!gc%eri#El$qkah##.#in!iFLDI#$nn..qaGieaAKei....n#hlIVF!sahsElalda%e..VnAL#%IqKnVdeerF...!ET.!

             131     140       150       160       170       180       190       200       210       220       230       240      249
             |--------+---------+---------+---------+---------+---------+---------+---------+---------+---------+---------+--------|
Enterococcus DAYIQQKENIKTKSEYIIINLKASSIKMDINDIYFFQ--TEYDHRVSMVGKNFKREFYGTLSKIEQLHPDLIRVHQSIIINKKYASKLNYKTHLLTMRDGTEVPVSRRYYTQVKALFLT
  Marinitoga NRIKQLFTNTENKTTKLAVEDEKEIIFLDFKDIYYFEFFDKTIHVFTFDNEYILKHFRKFSELSKNLPKNFVRIHKSYIVNLDYIEKYIKELSSLELKNKKILPIGKTYLRHVKKI
   Consensus #aiiQkeNienKsekia!#deaeiIk$DinDIY%F#..detdHrfsmdnenilreFrgflelie#Lhk#l!R!HqSiI!NldYaeKlnkelhlLe$r#gkelP!grrYlrqVKai...
```
