# Supplementary material for: Quorum sensing in thermophiles: prevalence of autoinducer-2 system
Source: BMC Microbiol. 2018 Jun 28;18:62. doi: 10.1186/s12866-018-1204-x (PMC6022435; doi:10.1186/s12866-018-1204-x)
Supplement: Supplementary file 5 — Multiple sequence alignment of ComA protein from Bacillus subtilis and thermophilic eubacteria by MultAlin. (PDF 120 kb) [file 12866_2018_1204_MOESM5_ESM.pdf]

```
                     1        10        20        30        40        50        60        70        80        90        100       110       120       130
                     |--------+---------+---------+---------+---------+---------+---------+---------+---------+---------+---------+---------+---------|
Bacillus             MKKILVIDDHPAYMEGTKTILETDSNLSVDCL-SPEPSEQFIKQHDFSSYDLILMDLNLGGEVNGMELSKQILQENPHCKIIVYYTGYEVEDYFEEAIRAGLHGAISKTESKEKITQYIYHVLNGEILVDF
Anoxybacillus        MARILIVDDHRLVCEGTKRMLECEQDFQVDFVTSAKEAEQMIEQR---TYDVFLLDWCMS-DVSGIELSKRILQKQPNAKIVIYTGYDIVPFLNYFIESGITGFVSKTASSEQLVTAIRCALRDEAVIPV
A.flavithermus       MARILIVDDHLLVCEGTRNVLEREGDFQVDITTSASEAEQMIEQC---AYDVCLLDWCMS-DMSGIELSKRILEKQPTAKIVIYTGYDIVPFLNYFIESGITGFVSKTASSEQLVTAIRCALRDEAVIPV
A.suryakundensis     MARILIVDDHLLVCEGTKNVLECEGDFQVDITTSASEAEQMIEQR---AYDVCLLDWCMP-DMSGIELSKRILEKQPNAKIVIYTGYDIVPFLNYFIELGITGFVSKTASSEQLITAIRCALRDEAVIPV
Parageobacillus      MIHILIVDDHRLVGEGTKNMLESEQDFQVELVTSGTEAEQIIEQR---TYDVYLLDWRMR-DINGIELSKRILQKQPNAKIVIYTGYDVVPFFNYLIESGVTSFISKTATRDQLITAIRCTLRDEAVIPV
G.icigianus          MIHILVVDDHRLVGEGTKNMLESEQDFQVELVTSGTEAEQMMEQQ---TYDVYLLDWRMP-DMNGIELGKRILQKQPNAKIVIYTGYDVAPFLNYLIESGITGFVSKTATSEQLITTIRCALRDEAVIPV
Marinithermus        MIRILLADDHALFRQGLRTLLEAEPEFRV--IGEAGNGREALRTALATRPDVILMDIQMP-ELDGVQATQEILREFPEARVIMLTMYRQDAYYVFEAVKAGARGYLLKDAQAEELIDAIRRVHQGEVLLDG
Oceanithermus        MIRILIADDHSLFRQGLRSLLEAEGDLRV--IGEAASGREALRAALETRPDIVLMDIQMP-GLDGVEATKEILKEFPEAKVIMLTMYRQDAYYVFEAVKAGARGYLLKDVDAEALVDAVRRVNEGEVLLDA
Thermus              MIRILLADDHALFRQGLKSLLEAEGDFKV--IGEAKDGREALRHALEAKPDVILMDIQMP-GLDGVQATQEILKEWPQARVIILTMYRQDAYYVFEAVKAGARGYLLKDTDAKELIEAIRRVAAGEVLLDA
T.aquaticus          MIRILLADDHALFRQGLKSLLEAEGDFRV--VGEAKDGWEALRHALEAKPDVILMDIQMP-GLDGVQATKAILQEWPGARVIILTMYRQDAYYVFEAVKAGARGYLLKDTDAKELIEAIRRVHAGEVLLDA
T.oshimai            MIRIVLADDHALFRQGLKSLLEAEGDFRV--VGEAKDGWEVLRHALEAKPDVILMDIQMP-GLDGVQATQAILKEWPEAKVIILTMYRQDAYYVFEAVKAGARGYLLKDTDAKELIGAIRRVHAGEVLLDA
T.igniterrae         MIRILLADDHALFRQGLKSLLEAEGDFRV--VGEAKDGWEALRHALEAKPDVILMDIQMP-GLDGVQATQAILKEWPAAKVIILTMYRQDAYYVFEAVKAGARGYLLKDTDASELIGAIRRVHAGEVLLDA
T.scotoductus        MIRVLLADDHALFRQGLKSLLEAEGDFRV--VGEAKDGWEALRHALEAKPDVILMDIQMP-GLDGVQATQAILKEWPQAKVIILTMYRQDAYYVFEAVKAGARGYLLKDTDASELIGAIRRVHAGEVLLDA
T.amyloliquefaciens  MIRVLLADDHALFRQGLKSLLEAEGDFRV--VGEAKDGWEAMRHALEAKPDVILMDIQMP-GLDGVQATQAILKEWPEARVIILTMYRQDAYYVFEAVKAGARGYLLKDTNASELIEAIRRVHAGEVLLDA
T.caliditerrae       MIRVILADDHALFRQGLKSLLEAEGDFRV--VGEAKDGWEAMRHALEAKPDVILMDIQMP-GLDGVQATQAILKEWPEARVIILTMYRQDAYYVFEAVKAGARGYLLKDTDASELIEAIRRVHAGEVLLDA
T.thermophilus       MIRVLLADDHALFRQGLKSLLEAEGDFRV--VGEAKDGWEALRHALETKPDVILMDIQMP-GLDGVQATQAILKEWPQAKVIMLTMYRQDAYYVFEAVKAGARGYLLKDVDARELVDAIRRVHAGEVLLDA
T.islandicus         MIRVLLADDHALFRQGLKSLLEAEGDFRV--VGEAKDGWEALRHALEAKPDVILMDIQMP-GLDGVQATQAILEEWPQAKVIILTMYRQDAYYVFEAVKAGARGYLLKDVDAKALIEAIRRVHAGEVLLDA
Meiothermus          MIRILIADDHALFRQGLKSLLEAEPDFKV--MGEAKDGREALRHALEAHPDIILMDIQMP-GLDGVQATQEILREWPQAKVIMLTMYRQDNYYVFEAVKAGARGYMLKDADAKELLEAIRQVHQGEVLLDA
M.cerbereus          MIRILLADDHALFRQGLKSLLEAEPDFKV--MGEAKDGREALRHALEAHPDIILMDIQMP-GLDGVQATQEILKEWPQAKVIMLTMYRQDSYYVFEAVKAGARGYMLKDADAKELLEAIRRVYQGEVLLDA
M.ruber              MIRILLADDHALFRQGLKSLLEAEPDFKV--MGEAKDGREALRHALEAHPDIILMDIQMP-NLDGVQATQEILKEWPQAKVIMLTMYCQDGYYVFEAVKAGARGYMLKDADAKELLEAIRRVYQGEVLLDA
M.chliarophilus      MIRILLADDHALFRQGLRSLLEAEPDFRV--IGEAKDGREALRHALEARPDIILMDIQMP-GLDGVQATQEILREWPQAKVIMLTMYRQDAYYVFEAVKTGARGYMLKDADAKELLEAIRRVQAGEVLLDA
M.silvanus           MIRIVLADDHALFRQGLRSLLEAEPDFRV--VGEAGDGREALRHVLEAKPDIVLMDIQMP-GLDGVQATQEILREWPQAKVIMLTMYRQDAYYVFEAVKAGARGYMLKDADAKDLLEAIRRVNNGEVLLDA
Thermomicrobium        RVLIVDDHDLFREGLRQLIETDPAIEV--VGEAANGQDALKMVAELQPDVVLMDINMP-GMDGIRATEAIVQQYRHVHVLMLTMYDDEEYVLHAIRAGARSYLVKNSKPEELLRAIHVAAEGGATIDP
A.geothermalis       MIKVLLVDDHEMVRLGVSAYLSAQPDMEV--IGEAEDGKKGVELALELRPDVILMDLVME-PMDGIAATQEIIHAWPEAKIIVVTSFLDDEKVFAALEAGATSYLLKTSKASEIADAIRATFHGQSVLEP
Geobacillus          MIKVLLVDDHEMVRLGVSAYLSAQPDMEV--IGEAEDGKRGVELALTLRPDVILMDLVME-PMDGIAATREIIGAWPEAKIIVVTSFLDDEKVFAALEAGATSYLLKTSKASEIADAIRATFHGQSVFEP
G.thermoleovorans    MIRVLLVDDHEMVRLGVSAYLSAQPDMEV--IGEAEDGKKGVELALQLRPDVILMDLVME-PMDGIAATREIIGAWPEAKIIVVTSFLDDENVFAALEAGATSYLLKTSKASEIADAIRATFHGQSVFEP
Chloroflexus           KVLVIDDHPLFRQGIRWSLESAPDIEV--VGEAENGQEAIKLTERLMPDIVLVDINLP-GLNGLEVARVIKRREPRTGIIVLSVYEDDDQLFQAIKVGAAAFTSKDISPDELVQMIREVARGRYLIND
A.thermarum            RIVLIDDHKLFREGIKRILDFEQDFEV--VAEGSDGDEALALVEQYKPDVVIMDINMP-HVNGVEATRQLIEAYPDTKVIILSIHDDESYVMHALQTGAMGYLLKEMDADALIEAVRVVAEGGSYLHP
Natranaerobius         RILLADDHALFRQGLKKILDMEEDLAV--CGEAQNGEEVVDKVKLLKPNIILMDINMP-LLNGVEATKYVKEVSPETKVIALTIHEDEEYLFEIVKEGAAGFLLKDVETPTLVQAIQEVNKGNSFIHP
T.carboxydivorans       ILVADDHALLRQGIKNVLALENDFAV--IGEAGDGEETLRKAAELAPDVILLDINMP-RLNGLEVTKRLRDSHPQIKVLILTIHDDDNYVVEVIKAGAAGYLLKDVEPSMLVAAIRAVYEGESFIYP
Pelotomaculum          KVLIVDDHPLVREGLVKILSTDDRINI--VGNAENGKQAVKIALEIKPDIILMDINLP-VLNGIEATRIIKGHLHETGIIALTIHDQEEYLFELIKAGISGYLLKDASPDLLIKTVLGVSRGESFIPP
Moorella                ILIADDHPLVREGIRKILELEPDLQV--VGEAATGQEALEMTRRYQPQVIIMDINMP-GLNGIEATKVIRQEWPQIAILALTIHDDEEYIIELLRSGVSGYVLKDIGAEELVQAVYQVARGNRVIHP
Caldanaerobacter     MIRLLVADDHALIRKGLVQLIELEKDIRV--ISQASNGEEAYRLAKELSPDIILMDINMP-VMNGITAAKKLKAEKHPSKILFLTIYNDKEYLVEALKLGVEGYILKDAEYDDLIKAVRTIYNGGVYIHP
T.thermocopriae      MIRLLVADDHALIRKGLVQLIEMENDIKV--ISQASNGEEAYKLAKKFMPDLILMDVNMP-VMNGIKAAKMLKEEKHPSKVLFLTIYNDREYLLEALKLGVEGYILKDAEYDDLIKAIRTIYNGGVYIHP
Thermoanaerobacteriu MINIMIADDHVLLRQGLKQIIELEEDMKV--SYQASDGEEAYNLAKNNSPDVILMDINMP-NVNGIKAAKMIKNDNPKNKIMFLTIYNDKEYLMEALKIGVEGYILKDADSDELIKAIRIISNGGVYIHP
T.saccharolyticum    MINIMIADDHVLLRQGLKQIIELEEDMKV--VYQASDGEEAYNLAKNNSPDVILMDINMP-NVNGIKAAKMIKNDNPKNKIMFLTIYNDKEYLMEALKIGVEGYILKDADSDELIKAIRIVSNGGVYIHP
T.xylanolyticum      MINIMIADDHVLLRQGLKQIIELEEDMKV--VYQASDGEEAYNLAKNNSPDIILMDINMP-NVNGIKAAKMIKSNDPKNKIMFLTIYNDKEYLMEALKIGVEGYILKDADSDELIKAIRIISNGGVYIHP
T.thermosaccharolyti MINILIADDHVLLRQGLKQIIELEEDMKV--IYQASDGEEAYEIAKKISPDIILMDINMP-NINGIKAAKMLKNDNPKNKIMFLTIYNDKEYLMEALKIGVEGYILKDADSDELIKAIRTISNGGVYIHP
Caldicellulosiruptor   KVLIVDDNPAVLDGLKIILELE-NFEV--VSLCTNAKEAIEFLEKKHADVVLMDIRMP-VMDGIEGTFNIKTKFPNVKVIILTTFCEEDYIKKSLSFGADGYILKSSDAKHIVNSILSVLDGKVVMDK
C.saccharolyticus      KVLIVDDNPAVLDGLKIILELE-NFDV--VSLCTNAKEAIEFLENRHVDVVLMDIRMP-VMDGIEGTFNIKTKFPNVKVIILTTFCEKDYIEKSLSFGADGYILKSSDAKHIVNSILSVLDGKVVMDK
Carboxydothermus       KVLLAEDHHLVRRGLALLINSQPDMEV--VGEVACGEDCCQAITAVSPDVVVLDLALP-DIPGLKVLERLKCLKPMPKVIILTMYEELEFLEEALKLGAKGYLIKRAAEEELIRAVREVARGEYYIHP
P.thermoglucosidasiu              MLGIQQLLEKEADLEV--VNTICHPARLEEEIIKHNPDVIVMDIRMK-NDNGIEWTKKITETYPTCKVVILSGYDYDEYIHAAYEAGAYAFVTKENSAFELANAIRQSHLGIKSFPA
Consensus            m..il..#Dh.l.r.Glk..le.#.#..V..vgea..g.ea........pD!il$Di.$p ..nGieatk.i....p..k!i.lt.y....y..ea.k.Ga.g%.lKd....el..a!r.v..g...i..

                     131      140       150       160       170       180       190       200       210       220  225
                     |--------+---------+---------+---------+---------+---------+---------+---------+---------+----|
Bacillus             AYFKQLMTQQKTKPAPSSQKEQDV----------LTPRECLILQEVEKGFTNQEIADALHLSKRSIEYSLTSIFNKLNVGSRTEAVLIAKSDGVL
Anoxybacillus        HFLRQLHRCE-GKEKMEENEQPAV----------LSHLEQELLMEVANGLCNKDIAKKHHTSQRTVERRLSRIFTKLHVSSRIEAVEKAKQLGLI
A.flavithermus       HVLRQLCRCG-AKENMDENGQLAV----------LSSLEQELLMEVANGLCNKDIAEKHHTSQRTIERHLSRIFTKLHVSSRIEAVEKAKQLGLI
A.suryakundensis     HVLRQLCRCG-AKENMDENGQLAV----------LSSLEQELLMEVANGLCNKDIAEKHHTSQRTIERHLSRIFTKLHVSSRIEAVEKAKQLGLI
Parageobacillus      HLLRQLRLRE-VRAKIEENGKQAA----------LSQLEQEILMEVANGLCNKDIAKKRYISQRSVERHLSHIFTKLHVSSRIEAVEKAKQLGLI
G.icigianus          HLLRQLHLCE-VKAKMEENEEHVA----------LSQFEQEILFEVANGLSNKDIAKKHYISQRSVERHISRIFTKLHVSSRIEAVEKARQLGLI
Marinithermus        ELAEQIIQDFRAKREALTTAPAAE----------LTEREVQILRLLAQGATNQEIADELGLSEKTVRNRLSEIFAKLHLNNRTQAALYAIREGL
Oceanithermus        ELAEQIIHDFRAKRDVLPQAHHAD----------LTDREVQILRLLAQGATNQEIADELGISEKTVRNRLSEIFSKLHLNNRTQAALYAIREGL
Thermus              ELAGQIIQDFKAKKEQAPGVH-AE----------LSEREVQILRLVAQGYTNLEIAAELSLSEKTVRNRLSEIFQKLHLNNRTQAALYAIREGL
T.aquaticus          ELAGRIIQDFRTRKEQSLPLH-AE----------LSEREIQILKLVAQGYTNLEIAAELQLSEKTVRNRLSEIFQKLHLNNRTQAALYAIREGL
T.oshimai            ELAGRIIQDFRAKKEGSAPLH-AE----------LSEREIQILKLVAQGYTNLEIAAELQLSEKTVRNRLSEIFQKLHLNNRTQAALYAIREGL
T.igniterrae         ELAGRIIQDFRAKKEASTPLH-AE----------LSEREIQILKLVAQGYTNLEIAAELQLSEKTVRNRLSEIFQKLHLNNRTQAALYAIREGL
T.scotoductus        ELAGRIIQDFRAKKESSLPLH-AE----------LSEREIQILKLVAQGYTNLEIAAELQLSEKTVRNRLSEIFQKLHLNNRTQAALYAIREGL
T.amyloliquefaciens  ELAGRIIQDFRAKKESSLPLH-AE----------LSEREIQILKLVAQGYTNLEIAAELQLSEKTVRNRLSEIFQKLHLNNRTQAALYAIREGL
T.caliditerrae       ELAGRIIQDFRAKKESSAPLH-AE----------LSEREIQILKLVAQGYTNLEIAAELQLSEKTVRNRLSEIFQKLHLNNRTQAALYAIREGL
T.thermophilus       ELAGHIIQDFRAKKEASQPLH-AE----------LSEREVQILRLVAQGYTNQEIAAELQLSEKTVRNRLSEIFQKLHLNNRTQAALYAIREGL
T.islandicus         ELAGRIIQDFRAKKEASAPLN-AE----------LTEREVQILKLLAQGYTNLEIASELGLSEKTVRNRLSEIFQKLHLNNRTQAALYAIREGL
Meiothermus          ELAEKIIQDFKAKQETAPKAH-AE----------LSEREVQILRLVAQGYTNLEIASELSLSEKTVRNRLSDIFQKLHLNNRTQAALYALREGL
M.cerbereus          EMAEQIIQDFKAKQEAIPKEH-AE----------LSEREVQILRLVAQGYTNLEIASELSLSEKTVRNRLSDIFQKLHLNNRTQAALYALREGL
M.ruber              EMAEQIIQDFKAKQETTPKAH-AE----------LSEREVQILRLVAQGYTNLEIAAELSLSEKTVRNRLSDIFQKLHLNNRTQAALYALREGL
M.chliarophilus      EMAEQIIQDFKSKNETAPKPH-AE----------LSDREVQILKLVAQGYTNLEIASELSLSEKTVRNRLSDIFQKLHLNNRTQAALYALREGL
M.silvanus           ELAEQIIQDFKAKRESVPKPH-AE----------LSEREVQILKLVAQGYTNLEIAGELSLSEKTVRNRLSEIFQKLHLNNRTQAALYALREGL
Thermomicrobium      DLAPILMREYQ-RLLTKAPTRGQD----------LSDRELTMLRLLAQGYNNRQIADALNLAESTVKNNLSALFQKLGVRDRTQAVIYAISHGLV
A.geothermalis       EVAGKVMQNRRRQRERLPH---EQ----------LTSREMEVLLLMAQGKTNQEMADELFISLKTVKVHVSNILAKLGVQDRTQAVIYAFRHGLV
Geobacillus          EVAGKVMRNYRKQEERLPH---EQ----------LTSREMEVLLLMAQGKTNQEMADELFISLKTVKVHVSNIFAKLGVQDRTQAVIYAFRHGLV
G.thermoleovorans    EVAGKVMRNYRKQEERLPH---EQ----------LTSREMEVLLLMAQGKTNQEMADELFISLKTVKVHVSNILAKLGVQDRTQAVIYAFRHGLV
Chloroflexus         AVLARPNVAARVLHQFRELAATEEEDSASL-FAPLTSREIEILDCIARGLSNKEIANELSISGQTVKNHITSILSKLQVNDRTMAVIVAIKKG
A.thermarum          KVTHNLVKEYRRLVAGEGTGQEKQKQAVRRPYHLLTRRECEVLQLLADGKSNKGIADALFISEKTVKNHVSNILQKLNVNDRTQAVVVAIKNG
Natranaerobius       TITHKLLGEFNRL-AQNSPRNIKSDEAVQSE---LTGREKEILELMAQGYTNKEISKELYISEKTVKNHVSNILRKMDVTDRTQAVITALKCGVV
T.carboxydivorans    TLAKRLLDETIR--RGEKKKQESAVQPARRGEERLTYRELEVLQLVAQGLSNSEIAQKLYLSEKTVKNHLTNIFRKINVTDRTQALIYA
Pelotomaculum        SLTAKMMAEFNRLTSISTGNANPQG---------LTRREVDVLRLLALGNNNRTIAKELFISEKTVKNHLTNIFQKLGVGDRTQAAIHA
Moorella             GVAQKVIGCIQKQETF-TPAPEPIP---------LTAREREILIHVSLGKSNREIARDLFISEKTVKNHLTHIFHKIGVSDRTQAALYALKHGL
Caldanaerobacter     SLMQEIEDSNEELLRKE-----------------LTPREIEILKLLSQGYSNKEIAQKLFLSEKTVKNHVYNIFRKLGVKDRTQAAI
T.thermocopriae      SLMEEIDNIDHENLKKD-----------------LTPREIEILKLISKGYSNKEIAQKLFLSEKTVKNHVYNIFKKLDVKDRTQAAI
Thermoanaerobacteriu SLIREIENLEKNECKKD-----------------LTDREFEILNLIAEGYSNKEIADKLFLSEKTVKNHVYNIFRKLDVKDRTQAAI
T.saccharolyticum    SLIREIENLEKNECKKD-----------------LTDREFEILNLIAEGYSNKEIADKLFLSEKTVKNHVYNIFRKLDVKDRTQAAI
T.xylanolyticum      SLIREIESLEKNECKKD-----------------LTDREFEILNLIAEGYSNKEIADKLFLSEKTVKNHVYNIFRKLDVKDRTQAAI
T.thermosaccharolyti SLVSEIENLDTDECKKE-----------------LTDREMQILSLIAEGYSNKEIADKLFLSEKTVKNHVYNIFRKLDVKDRTQAAI
Caldicellulosiruptor EIALYISDVLKRTSKQLLEKTQN-----------LTERELEIAKLISQGYSNKEIARMLFISEGTVRNYITSILQKLNLKNRTQ
C.saccharolyticus    EIALYVSDVLKRTSKQLFEKTQN-----------LSERELEIAKLISQGYSNKEIARMLFISEGTVRNYITSILQKLNLKNRTQ
Carboxydothermus     SLAGKMISRNALAAKCG-----------------LSPREMEVLVYWGKGLANEEIAKKLFISVKTVETHKTRIKEKLNLKTRADVV
P.thermoglucosidasiu DRLSNYHPP-------------------------LTNVELKILQFISEDRTNAEISELLNISKRTVEHHVSSILRKLDVDSRVGAVVKAMKLGIL
Consensus            .l................................Lt.rE.e!L.l.a.G..N.#Ia..l.iSektV.nh.s.If.K$.v..Rtqa...a...g..
```
