# Supplementary figures and images for: Quorum sensing in thermophiles: prevalence of autoinducer-2 system
Source: BMC Microbiol. 2018 Jun 28;18:62. doi: 10.1186/s12866-018-1204-x (PMC6022435; doi:10.1186/s12866-018-1204-x)

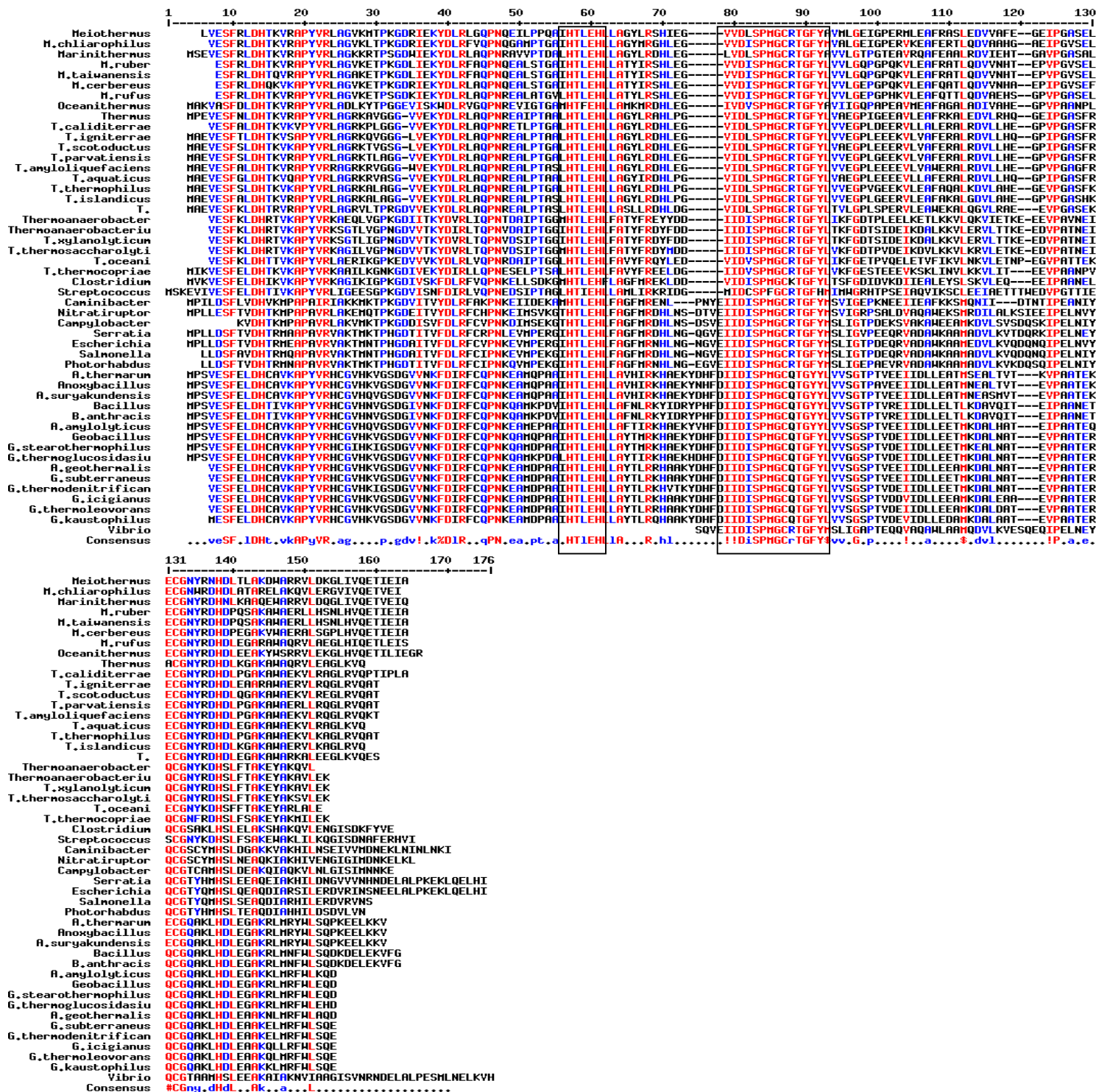

Supplement: Supplementary file 6 — Multiple sequence alignment of LuxS protein from mesophilic and thermophilic eubacteria by MultAlin. Invariant residues are highlighted in red colour. The conserved HTLEH motif and other conserved residues are highlighted within boxes. (PDF 111 kb) [file 12866_2018_1204_MOESM6_ESM.pdf]

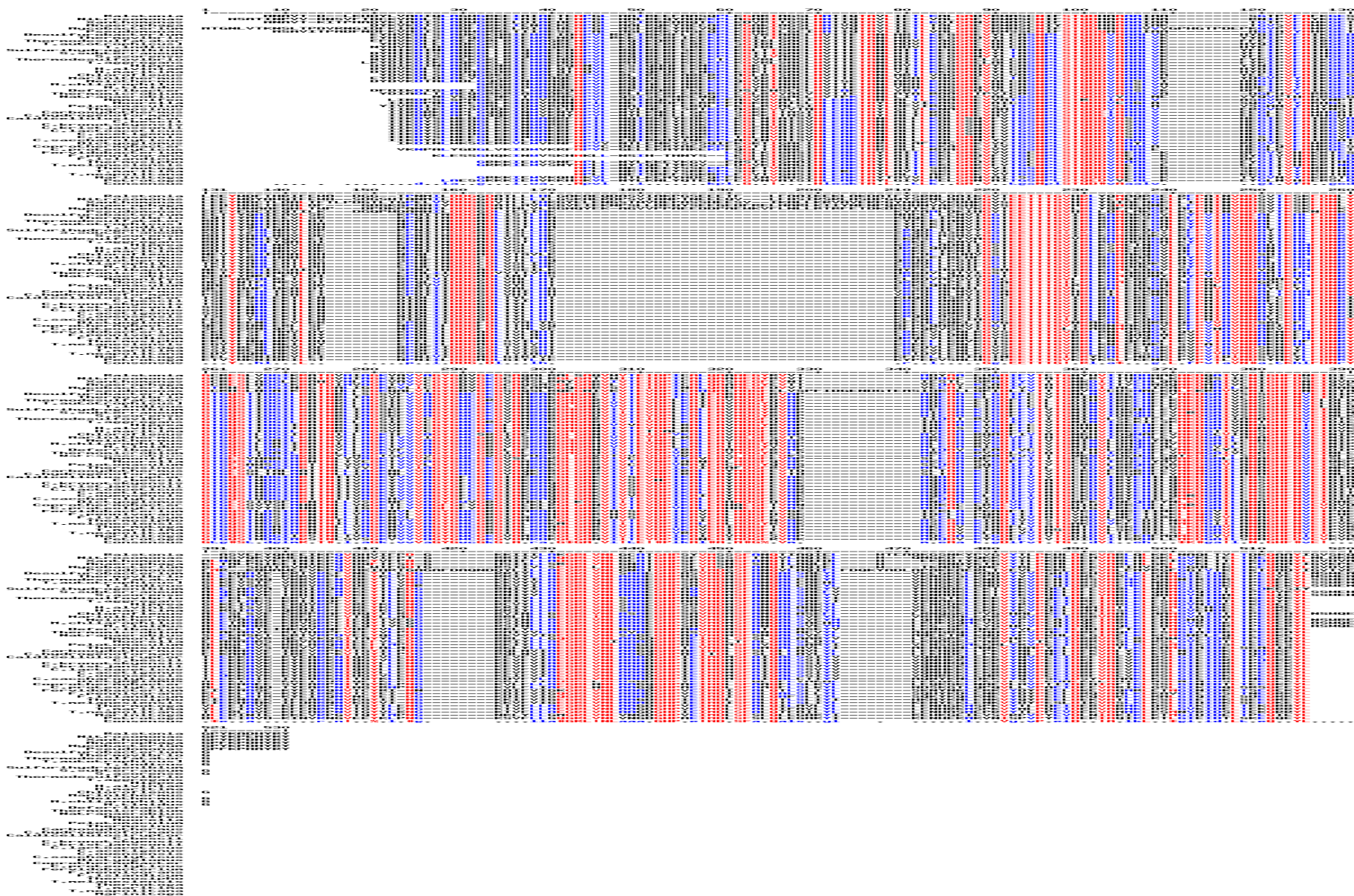

Supplement: Supplementary file 9 — Multiple sequence alignment of SAH hydrolase from thermophilic and mesophilic eubacteria by MultAlin. High conservation among SAH hydrolases has been observed. (PDF 274 kb) [file 12866_2018_1204_MOESM9_ESM.pdf]

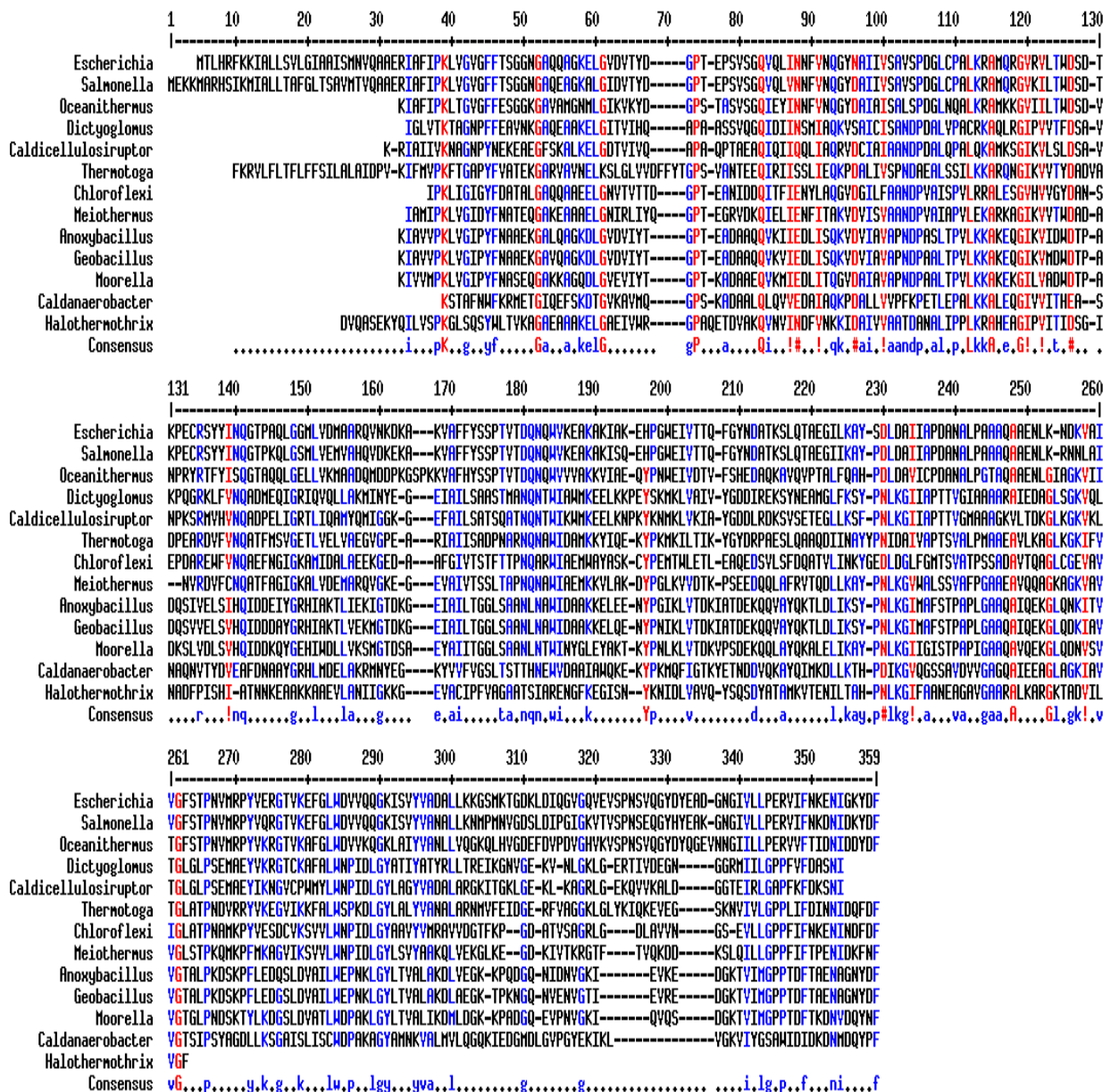

Supplement: Supplementary file 11 — Multiple sequence alignment of LsrB from mesophilic and thermophilic eubacteria by MultAlin. (PDF 105 kb) [file 12866_2018_1204_MOESM11_ESM.pdf]
