# Supplementary material for: Quorum sensing in thermophiles: prevalence of autoinducer-2 system
Source: BMC Microbiol. 2018 Jun 28;18:62. doi: 10.1186/s12866-018-1204-x (PMC6022435; doi:10.1186/s12866-018-1204-x)
Supplement: Supplementary file 7 — Multiple sequence alignment of MTA/SAH nucleosidase from mesophilic and thermophilic eubacteria by MultAlin. (PDF 88 kb) [file 12866_2018_1204_MOESM7_ESM.pdf]

```
                     1       10        20        30        40        50        60        70        80        90        100       110       120       130
                     |--------+---------+---------+---------+---------+---------+---------+---------+---------+---------+---------+---------+---------|
Escherichia                           MKIGIIGAMEEEVTLLRDKIENRQTISLGGCEIYTGQLNGTEVALLKSGIGKVAAALGATLLLEHCKPDVIINTGSAGGLAPTLKVGDIVVSDEARYHDADVTAFGYEYGQLP
Anoxybacillus                         MKVAIIGAMEEEVAILREKIENRNDTTIANCEFYTGTLHGVDVVLLKSGIGKVNAAMSTTILLERFQPDVVINTGSAGGFLASLNVGDIVISTEAAHHDVDVTAFGYAYGQVP
A.amylolyticus                        MKVAIIGAMEEEVAILREKIENRNDTTIANCEFYTGTLHGVDVVLLKSGIGKVNAAMSTTILLERFQPDVVINTGSAGGFLASLNVGDIVISTEAAHHDVDVTAFGYAYGQVP
A.thermarum                           MKIAIIGAMEEEVAILREKIANRTETTVANCSFYSGTLDGADVVLLKSGIGKVNAAMSTTILLERFAPDVVINTGSAGGFAPSLNVGDIVISTEVVHHDVDVTAFGYAYGQVP
A.flavithermus                        MNIAIIGAMEEEVAILREKIANRTETTVANCSFYSGTLDGANVVLLKSGIGKVNAAMSTTILLERFAPDVVINTGSAGGFAPSLNVGDIVISTEVVHHDVDVTAFGYAYGQVP
A.suryakundensis                      MKIAIIGAMEEEVAILREKIADRTETTVANCAFYSGTLDGANVILLKSGIGKVNAAMSTTILLERFAPDVVINTGSAGGFAPSLNVGDIVISTEVVHHDVDVTAFGYAYGQVP
A.geothermalis                        MKVAIIGAMEEEVAILREKMDNRQDTVAANCEFYTGTLNGVDVILLKSGIGKVNAAMSTTILLERFRPDYVINTGSAGGFSASLNVGDIVISSEVVHHDVDVTVFGYEYGQVP
G.subterraneus                        MKAAIIGAMEEEVAILRSRMDGREEVVIAGCEFSIGRLDGVEAVLLKSGIGKVNAAMGTTLLLDRFRPDVVINTGSAGGFLPSLRVGDLVISDEVVHHDVDVTAFGYAYGQVP
G.kaustophilus                        MKAAIIGAMEEEVAILRSRMEGREETVIAGCEFSEGCLDGVQAVLLKSGIGKVNAAMGTTLLLDHFRPDFVINTGSAGGFLPSLRVGDLVISEEVVHHDVDVTAFGYAYGQVP
G.thermocatenulatus                   MKAAIIGAMEEEVAILRSRMEGREETVIAGCEFSKGSLDGVEAVLLKSGIGKVNAAMGTTLLLDRFRPDFVINTGSAGGFLPSLRVGDLVISEEVVHHDVDVTAFGYAYGQVP
G.caldoxylosilyticus                  MKVAIIGAMEEEVTILRDQMEGREETVIANCEFSTGQINGVDVILLKSGIGKVNAAMATAILLERFRPNYVINTGSAGGFLAALNVGDIVISSEVVHHDVDVTAFGYEYGQVP
G.stearothermophilus                  MKVAIIGAMEEEVTILRDQMEGREETVIANCEFSTGRINGVDVILLKSGIGKVNAAMATAILLERFRPNYVINTGSAGGFLATLNVGDIVISSEVVHHDVDVTAFGYEYGQVP
G.thermoglucosidasiu                  MKIAIIGAMEEEVAILRDKMEERKETIIANCEFSLGRLNGADVILLKSGIGKVNAAMATAILLERFRPNYVINTGSAGGFLSTLNVGDVVISTEVVHHDVDVTVFGYEYGQVP
T.kivui                                KIGLIGAMEEEVNILKNEMSLKEIVTKGKLDFYIGSLNGSDIVVVQSGIGKVNAAMAAQILISNFNIDILINTGVAGGIKPGVKVGDIVISSDAMEYDIDVTAFGYELGIIP
T.thermocopriae                        KIGLIGAMEEEINILKDEMTLKEIVKKAKLDFYVGSLKSADIVIVRSGIGKVNAAMATQILVSDFNVDVLINTGVAGGIKPDIKVGDIVISSDTMEYDIDVTAFGYEPGIIP
T.saccharolyticum                      KIGFIGAMEEEVHLLKEAIVNKLTINRADMDFFSGILNGVDAVVVKSGIGKVNAAIATQILISEFKVDCIINTGVAGGLKKGINVGDIVISSDAIEHDFDTTAFGDELGVIP
T.aotearoense                          KIGFIGAMEEEVELLKEAIVNKLTINRADMDFFSGILNGVDAVVVKSGIGKVNAAIATQILISEFKVDCIINTGVAGGLKKGINVGDIVISSDAIEHDFDTTAFGDELGVIP
T.xylanolyticum                        KIGFIGAMEEEVELLKEAIVNELTINRADMDFFSGIINGVDAVVVKSGIGKVNAAIATQILISEFKVDCIINTGVAGGLKKGINVGDIVISSDAIEHDFDTTAFGDELGVIP
T.thermosaccharolyti                   KIGLIGAMEEEIDILKHFISNVEIINRADMVFYKGILNGLDTVLVRSGIGKVNAAIAAQILISEFNVDYIINTGVAGGIKSGINVGDIVISSDAIEHDFDTTAFGDELGVIP
T.oceani                                IGIIGAVDREIALFCEELKDKKTITKASLTFFTGTLEGRDAVVVKCGVGKVNAAICTQVMIDYFAPSAIICTGVAGALREDLDIGDIVISRDVVQHDVDGTAFGHELGEIP
Nitratiruptor        MKIAIMGAMIEEIEPILSRLDSTFSTDEPLEYIIEHMQNIKLHYIAGNRYFEAKYKGHEIIVAYSKIGKVYASLTANVLIQHFKVEKLLFSGVAGAISEDLHIGDLIMAKRLCQHDLDITAFGHPYGYVP
Caminibacter                            IGILCAMREELEPILEEVEVKEVVEYGKNKFYLAKFDNKDLVLAYSKIGKVNSATTATIMIEKFKAKKILFSGVAGAIDEDLKIGDLIIATKTCQHDIDLTVFGYPYGYIP
Meiothermus                                     EAQALRRLLSLKETLD-GPWIIHQGSLGRYPVVLVETGVGKVAASAAVAYAKVRFNPAQAFWVGVAGALNPTLKTMDLILAQDAVQYDVDITAFGRAPGEL-
M.chliarophilus                         MALFAAEGAEARALREALGLNESLQ-GPKPIFRGQVGGHEVLLVETGVGKVAAASAVTYAQARFNPRWSLWAGVAGGLNPRLHSLDVLVAHDAVQWDVDITAFGRQPGEL-
M.silvanus                                                                HRGIFADHEVVLIEAGVGKVAAASAVTYAQARFEPKIAIWAGVAGALNPKLHALDVLIAHDAVQWDVDITAFGRKPGEL-
Thermus                                   FFAAEPEEASALREALGAGEALE-APFPLHRG----EGVLVAETGVGKVAAALAVAHVLTRFRPSESFFLGVAGALDPSLRALDLLLAEKAVQWDVDLTPFGRKPGET-
T.islandicus                                                               RG----RGVLVAETGVGKVAAAMAVAHVLTRFAPEASFFLGVAGALDPSLKALDLLLAEQAVQWDVDLTPFGRLPGET-
Thermotoga                                                                  GVVGRNEVVVSYGFIGKVEAALVTQAFLDRFNIDTVFLTGNAGGLE-GVEVGDVVIGDSYVEYDFETAL---------
T.neapolitana                                                               GVVGRNEVVVSYGFIGKVEAALVTQAFLDRFNIDAVFLTGNAGGLE-GVEVGDVVIGDSYVEYDFETAL---------
T.maritima                                                                  GVVGRNEVVVSYGFIGKVEAALVTQAFLDRFNIDAVFLTGNAGGLE-GVEVGDVVIGDSYVEYDFETAL---------
Thermosipho                                                                 GLIGVNEVLLVYGLIGKVESAMMAQALIDRFKPKYFIHCGSAGAINKERKIGDIVCGTEYIEHDISFRE---------
Fervidobacterium                             LKEEIIGVFREMLPLLENGELVKRNFSRGIVGGNEVVTIYGFVGKVESAMIAQAIIDKFRPSYIIHCGSAGAIHPQLKIGDLVCGTVFYEHD--FQS---------
F.nodosum                                    LKEEIVGVFRELLPLLENGELIKRNFSRGIIGGNEVVTIYGFIGKVESAMITQAIIDKFNPKYIIHCGSAGAIDPNLKIGDIVCGTTYYEHD--FQG---------
Consensus                             ..........ee.......................G...g.evv....g!GKV.aA........rF.p...i.tG.AGgl...l..gD.vi....v.hD.d.t.fg...g...

                     131     140       150       160       170       180       190       200       210       220       230       240       250       260
                     |--------+---------+---------+---------+---------+---------+---------+---------+---------+---------+---------+---------+---------|
Escherichia          GCPAG-FKADDKLIAAAEACIAE-LN-LNAVRGLIVSGDAFINGSVGLAKIRHNFPQAIAVEMEATAIAHVCHNFNVPFVVVRAISDVADQQSHLSFDEFLAVAAKQSSLMVESLVQKLAH--G
Anoxybacillus        GLPAR-YVSDEKLVAIAEKSAAN-IRGVQVAKGLIATGDSFMHDPARVEFVRTQFPELCAVEMEAAAIAQVCHQFGVPFVVIRALSDIAGKESNVSFEQFLHKAALHSSELVQAMVNEL
A.amylolyticus       GLPAR-YVSDEKLVAIAEKSAAN-IRGVQVAKGLIATGDSFMHDPARVEFVRTQFPELCAVEMEAAAIAQVCHQFGVPFVVIRALSDIAGKESNVSFEQFLHKAALHSSELVQAMVNEL
A.thermarum          GMPAR-YKADERLVQAAETSAAH-IRDIQVAKGLIATGDSFMNDPARVDFVRTQFPDLYAVEMEAAAIAQVCHQFGVPFVVIRALSDIAGKESNVSFEQFLQKAALHSSELVQWMVNEL
A.flavithermus       GMPAR-YAADERLIQAAETSAAH-IRDIQVAKGLIATGDSFMHDPARVDFVRTQFPDLYAVEMEAAAIAQVCHQFNVPFVVIRALSDIAGKESNVSFEQFLQKAALHSSELVQLMVNELFV--RTQFPDL
A.suryakundensis     GMPAR-YVADERLVQAAEASAAH-IRDIQVAKGLIATGDSFMHDPARVDFVRTQFPDLYAVEMEAAAIAQVCHQFAVPFVVIRALSDIAGKESNVSFEQFLQKAALHSSELVQLMVNEL
A.geothermalis       GMPAR-YAADAKLVAIAEASAER-IQEIQVVKGLIATGDSFMNDPERVAFVREKLTDLHAVEMEGAAIAQVCFQFGVPFVVIRALSDIAGKESNVSFEQFLHKAALHSSELVQMMVDELKH
G.subterraneus       GLPAR-YRADETLVEAAKQAAAR-LDGLQAAVGLIATGDSFMNDPKRVEFVRGQFPELCAVEMEAAAIAQVCVQFETPFVIIRALSDIAGEESDVSFEQFLETAAKHSAELVLSMLAVVQK
G.kaustophilus       GLPAR-YRADEALVEAAKQAAAR-LDGLQAVTGLIATGDSFMNDPKRVEFVRGQFPELCAVEMEAAAIAQVCVQFGTPFVIVRALSDIAGEESDVSFEQFLETAAKHSAELVLSML
G.thermocatenulatus  GLPER-YRADEALVEAAKQAAAR-LDGLQAVTGLIATGDSFMNDPTRVEFVRGQFPELCAVEMEAAAIAQVCVQFGTPFVIIRALSDIAGEESNVSFEQFLETAAKHSAELVLSML
G.caldoxylosilyticus GMPAR-YQADKTLIDIAKRSAQE-INDVQVVTGLIATGDSFMNDPARVEFVRGKFPELCAVEMEAAAIAQVCTQFAVPFVIIRALSDIAGKESDISFEQFLDTAAKHSARLVASMLSLL
G.stearothermophilus GMPAR-YQANKTLIDIAKRGAQE-ISDVQVVTGLIATGDSFMNDPERVEFVRGKFPELCAVEMEAAAIAQVCTQFAVPFVIIRALSDIAGKESDISFEQFLDTAAKHSARLVYSMLSLL
G.thermoglucosidasiu GMPAR-YKADVTLVKIAEQNAKQ-IKDIQVVKGLIATGDSFMSDPARVEFVRSKFPELCAAEMEAAAIAQVCTQFAVPFVIIRALSDIAGKESDVSFEQFLDTAAKHSADLVLSIVSSL
T.kivui              RMEESIFRADGKLVELAKKTAENIAESD-VYVGRIISGDKFVSSKEEAKRLGEMF-NAYAVEMEGAAIGHVAYLNNIPFVIIRSISDNANDEASVDFSAFVEHAANISSKIVKEMIKLL
T.thermocopriae      RMEESIFKADEKLVELAKKVAKNIVEGR-AYVGRIISGDKFVSSKEEAKRLGKMF-NAYAVEMEGAAIAHVAFLNSIPFVIIRSISDNANEEASIDFNTFIEQAAKSSTKIVSEMVKLL
T.saccharolyticum    RMKTSVFKADEYLIDVAYKAANDNIDGK-AYIGRIVSGDKFISSKDEALKLGKLF-NALAVEMEGAAIAHTSYLNNVPFVVIRSISDNADGNAAKDFSQFVKEAAIVSSNIVKEMIN
T.aotearoense        RMKTSVFKADEYLIDVAYKAANDNIDGK-VYIGRIVSGDKFISSKDEALKLGKLF-NASAVEMEGAAIAHTSYLNNVPFVVIRSISDNADGNAAKDFSQFVKEAAIVSSNIVKEMIN
T.xylanolyticum      RMKTSVFKADEYLIDVAYKAANDNIDGK-AYIGRIVSGDKFISSKDEALKLGRLF-NALAVEMEGAAIAHTSYLNNIPFVIIRSISDNADGNATKDFSQFVKEAAIVSSNIVKEMIN
T.thermosaccharolyti RMKTSVFKADKYLIDIALKAANDNIDGK-AYVGRIVSGDRFVSSKEEARRLGQQF-NAYAVEMEGAAIAHTAYLNNIPFVIIRSISDNADGNATSDFNLFVKKASIVSSNLVKEMINMI
T.oceani             NLGIKAFTADETLVQIAQEVARKQVKGRRAITGRILSGDQFISSKEKVKFLRSFF-DGACVEMEGGAIGQVCYLNKVPFLIIRSISDRADGKAVSVYETFADEAARNSSHIVLGIIKLYEGFVRSKFPDL
Nitratiruptor        EGKVY-VESDPVLREIAKDVAKQ--MQVPLKEGTIATGDQFIADPKRKEWIQKTF-DADALEMEGAAVAVVCDAFDIPFFILRSISDAADMDASFDFDTFLKSSSKRSAEFILKMVDAIIQ
Caminibacter         ESEVY-FECDKSLNSIAKSVAKK--LNLKLKEGIIASGDQFIHSKEKKEWIKKTF-NASAIEMEGGAVGCVCFTLNIPFFMLRAISDSAEEGAGVDFDEFLEESSKVSAKFLIEMLKE
Meiothermus          ATGERFIPADAGLTSKVLRTAQA--MGLPIQLGRIASADRFLAHRGEAEEVRRVF-AADAVEMEGAAALWTARRLGLPMALLRAITDQAGSEAPIAFETFLERASERLAQLISQVL
M.chliarophilus      ATGERFVRADIDLSTRVYRAALE--LGFPVHWGRVASGDRFLAHPADAARLREVF-AADAVEMEGAAALWTAKDLALPMALVRTISDGADDDAHESFEMFLEQASERLAQVVRKVLQQ
M.silvanus           ASGERFVEADAELSTQLYRAALA--LNLPVHWGRIASGDRFLADPNEAQRLREVF-AADAVEMEGAAALWTAGRLGVPMALVRAISDGAGDEATVNFQEFLEVASRR
Thermus              AFGVAFFPSDPALLARAEKAALA--LGLPFRRGVVATGDRFLAQREEAERLRALH-GADAVEMEGAAALMVAWRFRHPMVLLRVVTDGAGEGAALDFQAFLREAARRLGLLARALVE
T.islandicus         ALGVRFFPSDPALLARVEEAAGR--LGLPHRLGVVATGDRFLADPKEAQGLRERF-GAQAVEMEGAAALMVAFRFRHPMALVRAVTDGAGEGAALDFQAFLREASRRLGLLARALV
Thermotoga           -GDEGIIVSGSEDLENKVIAYS----NREIKTGLIASGDAFVTTKEKAEEIKRRT-GALCVDMDSAAVAKVCHENEKKFLAIKTIVDVCGRETEEEF
T.neapolitana        -GDEGIIVSGSEDLEDKVIAYS----NREIKTGLIASGDAFVTVKEKAEEIKRRT-GALCVDMDSAAVAKVCHENEKKFLAIKTIVDICGRETEEEFRKNYERYGFLSNLILLDVLKK
T.maritima           -GDEGIIVSGSEDLEDKVIAYS----NREIKTGLIASGDAFVTVKEKAEEIKRRT-GALCVDMDSAAVAKVCYENEKKFLAIKTIVDICGRETEEEF
Thermosipho          -KNVRIFKASDVLMERIYDIFN------DIKFGVIASGDVFVDSEEEKERIYRET-NAEVVDMDSAAIAKVCAENDINFCALKIVVDSSMEGSEIEIKRNLKRLAPFPSAIIAEMLEK
Fervidobacterium     -YTTLKIESSSKLLEKISDVYD------KINFGPIVSGDVIVSDRETKTKLYEKY-GAFAVDMDSAAIAKVCYQNGVEFCALKVIVDTSEEQAQIEYEQNFRKFSSLPSAIISELLDK
F.nodosum            -GNIMPIEASNVLLEKIADVYK------YVAFGPMVSGDLIVSDKKMKEMLYDKY-KALAVDMDSAAISKVCKENGVEFCSLKVIVDTSEEQAHTEYEQNFRKFSSLPSTIVSELLDK
Consensus            ........ad..l.......a...........G.!asGD.F....e.ae.....f..a.a!#M#gaAia.vc.....pf...r.isD.a...a...%..fl..a................

                     261     270       280       290       300       310       321
                     |--------+---------+---------+---------+---------+---------+|
Escherichia
Anoxybacillus
A.amylolyticus
A.thermarum
A.flavithermus       YAVEMEAAAIAQVCHQFGVPFVVIRALSDIAGKESNVSFEQFLQKAALHSSELVQWMVNEL
A.suryakundensis
A.geothermalis
G.subterraneus
G.kaustophilus
G.thermocatenulatus
G.caldoxylosilyticus
G.stearothermophilus
G.thermoglucosidasiu
T.kivui
T.thermocopriae
T.saccharolyticum
T.aotearoense
T.xylanolyticum
T.thermosaccharolyti
T.oceani             CAVEMEAAAIAQVCTQFAVPFVIIRALSDIAGKESDVSFEQFLDTAAKHSADLVLSIVSSL
Nitratiruptor
Caminibacter
Meiothermus
M.chliarophilus
M.silvanus
Thermus
T.islandicus
Thermotoga
T.neapolitana
T.maritima
Thermosipho
Fervidobacterium
F.nodosum
Consensus
```
