# Supplementary material for: Quorum sensing in thermophiles: prevalence of autoinducer-2 system
Source: BMC Microbiol. 2018 Jun 28;18:62. doi: 10.1186/s12866-018-1204-x (PMC6022435; doi:10.1186/s12866-018-1204-x)
Supplement: Supplementary file 8 — Multiple sequence alignment of MTA/SAH nucleosidase from thermophilic eubacteria by MultAlin. There is no conservation among Pfs protein sequences. (PDF 138 kb) [file 12866_2018_1204_MOESM8_ESM.pdf]

```
                      1       10        20        30        40        50        60        70        80        90       100       110       120       130
                      |--------+---------+---------+---------+---------+---------+---------+---------+---------+---------+---------+---------+---------|
         Meiothermus                              EAQALRRLLSLKETLD-GPWIIHQGSLGRYPVVLVETGVGKVAASAAVAYAKVRFNPAQAFWYGVAGALNPTLKTMDLILAQDAVQYDVDITAFGRAPGEL-
     M.chliarophilus                     MALFAAEGAEARALREALGLNESLQ-GPKPIFRGQVGGHEVLLVETGVGKVAAASAVTYAQARFNPRWSLWAGVAGGLNPRLHSLDVLVAHDAVQWDVDITAFGRQPGEL-
          M.silvanus                                                        HRGIFADHEVVLIEAGVGKVAAASAVTYAQARFEPKIAIWAGVAGALNPKLHALDVLIAHDAVQWDVDITAFGRKPGEL-
             Thermus                       FFAAEPEEASALREALGAGEALE-APFPLHRG----EGVLVAETGVGKVAAALAVAHVLTRFRPSESFFLGVAGALDPSLRALDLLLAEKAVQWDVDLTPFGRKPGET-
        T.islandicus                                                         RG----RGVLVAETGVGKVAAAMAVAHVLTRFAPEASFFLGVAGALDPSLKALDLLLAEQAVQWDVDLTPFGRLPGET-
       Nitratiruptor  MKIAIMGAMIEEIEPILSRLDSTFSTDEPLEYIIEHMQNIKLHYIAGNRYFEAKYKGHEIIVAYSKIGKVYASLTANVLIQHFKVEKLLFSGVAGAISEDLHIGDLIMAKRLCQHDLDITAFGHPYGYVP
        Caminibacter                     IGILCAMREELEPILEEVEVKEVVEYGKNKFYLAKFDNKDLVLAYSKIGKVNSATTATIMIEKFKAKKILFSGVAGAIDEDLKIGDLIIATKTCQHDIDLTVFGYPYGYIP
        Anoxybacillus                  MKVAIIGAMEEEVAILREKIENRNDTTIANCEFYTGTLHGVDVVLLKSGIGKVNAAMSTTILLERFQPDVVINTGSAGGFLASLNVGDIVISTEAAHHDVDVTAFGYAYGQVP
      A.amylolyticus                 MKVAIIGAMEEEVAILREKIENRNDTTIANCEFYTGTLHGVDVVLLKSGIGKVNAAMSTTILLERFQPDVVINTGSAGGFLASLNVGDIVISTEAAHHDVDVTAFGYAYGQVP
         A.thermarum                 MKIAIIGAMEEEVAILREKIANRTETTVANCSFYSGTLDGADVVLLKSGIGKVNAAMSTTILLERFAPDVVINTGSAGGFAPSLNVGDIVISTEVVHHDVDVTAFGYAYGQVP
      A.flavithermus                 MNIAIIGAMEEEVAILREKIANRTETTVANCSFYSGTLDGANVVLLKSGIGKVNAAMSTTILLERFAPDVVINTGSAGGFAPSLNVGDIVISTEVVHHDVDVTAFGYAYGQVP
    A.suryakundensis                 MKIAIIGAMEEEVAILREKIADRTETTVANCAFYSGTLDGANVILLKSGIGKVNAAMSTTILLERFAPDVVINTGSAGGFAPSLNVGDIVISTEVVHHDVDVTAFGYAYGQVP
      A.geothermalis                 MKVAIIGAMEEEVAILREKMDNRQDTVAANCEFYTGTLNGVDVILLKSGIGKVNAAMSTTILLERFRPDYVINTGSAGGFSASLNVGDIVISSEVVHHDVDVTVFGYEYGQVP
      G.subterraneus                 MKAAIIGAMEEEVAILRSRMDGREEVVIAGCEFSIGRLDGVEAVLLKSGIGKVNAAMGTTLLLDRFRPDVVINTGSAGGFLPSLRVGDLVISDEVVHHDVDVTAFGYAYGQVP
      G.kaustophilus                 MKAAIIGAMEEEVAILRSRMEGREETVIAGCEFSEGCLDGVQAVLLKSGIGKVNAAMGTTLLLDHFRPDFVINTGSAGGFLPSLRVGDLVISEEVVHHDVDVTAFGYAYGQVP
 G.thermocatenulatus                 MKAAIIGAMEEEVAILRSRMEGREETVIAGCEFSKGSLDGVEAVLLKSGIGKVNAAMGTTLLLDRFRPDFVINTGSAGGFLPSLRVGDLVISEEVVHHDVDVTAFGYAYGQVP
G.caldoxylosilyticus                 MKVAIIGAMEEEVTILRDQMEGREETVIANCEFSTGQINGVDVILLKSGIGKVNAAMATAILLERFRPNYVINTGSAGGFLAALNVGDIVISSEVVHHDVDVTAFGYEYGQVP
G.stearothermophilus                 MKVAIIGAMEEEVTILRDQMEGREETVIANCEFSTGRINGVDVILLKSGIGKVNAAMATAILLERFRPNYVINTGSAGGFLATLNVGDIVISSEVVHHDVDVTAFGYEYGQVP
G.thermoglucosidasiu                 MKIAIIGAMEEEVAILRDKMEERKETIIANCEFSLGRLNGADVILLKSGIGKVNAAMATAILLERFRPNYVINTGSAGGFLSTLNVGDVVISTEVVHHDVDVTVFGYEYGQVP
             T.kivui                  KIGLIGAMEEEVNILKNEMSLKEIVTKGKLDFYIGSLNGSDIVVVQSGIGKVNAAMAAQILISNFNIDILINTGVAGGIKPGVKVGDIVISSDAMEYDIDVTAFGYELGIIP
     T.thermocopriae                  KIGLIGAMEEEINILKDEMTLKEIVKKAKLDFYVGSLKSADIVIVRSGIGKVNAAMATQILVSDFNVDVLINTGVAGGIKPDIKVGDIVISSDTMEYDIDVTAFGYEPGIIP
   T.saccharolyticum                  KIGFIGAMEEEVHLLKEAIVNKLTINRADMDFFSGILNGVDAVVVKSGIGKVNAAIATQILISEFKVDCIINTGVAGGLKKGINVGDIVISSDAIEHDFDTTAFGDELGVIP
       T.aotearoense                  KIGFIGAMEEEVELLKEAIVNKLTINRADMDFFSGILNGVDAVVVKSGIGKVNAAIATQILISEFKVDCIINTGVAGGLKKGINVGDIVISSDAIEHDFDTTAFGDELGVIP
      T.xylanolyticum                 KIGFIGAMEEEVELLKEAIVNELTINRADMDFFSGIINGVDAVVVKSGIGKVNAAIATQILISEFKVDCIINTGVAGGLKKGINVGDIVISSDAIEHDFDTTAFGDELGVIP
T.thermosaccharolyti                  KIGLIGAMEEEIDILKHFISNVEIINRADMVFYKGILNGLDTVLVRSGIGKVNAAIAAQILISEFNVDYIINTGVAGGIKSGINVGDIVISSDAIEHDFDTTAFGDELGVIP
            T.oceani                   IGIIGAVDREIALFCEELKDKKTITKASLTFFTGTLEGRDAVVVKCGVGKVNAAICTQVMIDYFAPSAIICTGVAGALREDLDIGDIVISRDVVQHDVDGTAFGHELGEIP
          Thermotoga                                                          GVVGRNEVVVSYGFIGKVEAALVTQAFLDRFNIDTVFLTGNAGGLE-GVEVGDVVIGDSYVEYDFETAL---------
       T.neapolitana                                                          GVVGRNEVVVSYGFIGKVEAALVTQAFLDRFNIDAVFLTGNAGGLE-GVEVGDVVIGDSYVEYDFETAL---------
          T.maritima                                                          GVVGRNEVVVSYGFIGKVEAALVTQAFLDRFNIDAVFLTGNAGGLE-GVEVGDVVIGDSYVEYDFETAL---------
         Thermosipho                                                          GLIGVNEVLLVYGLIGKVESAMMAQALIDRFKPKYFIHCGSAGAINKERKIGDIVCGTEYIEHDISFRE---------
    Fervidobacterium                          LKEEIIGVFREMLPLLENGELVKRNFSRGIVGGNEVVTIYGFVGKVESAMIAQAIIDKFRPSYIIHCGSAGAIHPQLKIGDLVCGTVFYEHD--FQS---------
           F.nodosum                          LKEEIVGVFRELLPLLENGELIKRNFSRGIIGGNEVVTIYGFIGKVESAMITQAIIDKFNPKYIIHCGSAGAIDPNLKIGDIVCGTTYYEHD--FQG---------
           Consensus                 ..........ee........................G.....evv....g!GKV.aA........rF.p...i..GvAGal...l..gD.vi....v.hD.d.t.fg...g...

                      131     140       150       160       170       180       190       200       210       220       230       240       250       260
                      |--------+---------+---------+---------+---------+---------+---------+---------+---------+---------+---------+---------+---------|
         Meiothermus  ATGERFIPADAGLTSKVLRTAQA--MGLPIQLGRIASADRFLAHRGEAEEVRRVF-AADAVEMEGAAALWTARRLGLPMALLRAITDQAGSEAPIAFETFLERASERLAQLISQVL
     M.chliarophilus  ATGERFVRADIDLSTRVYRAALE--LGFPVHWGRVASGDRFLAHPADAARLREVF-AADAVEMEGAAALWTAKDLALPMALVRTISDGADDDAHESFEMFLEQASERLAQVVRKVLQQ
          M.silvanus  ASGERFVEADAELSTQLYRAALA--LNLPVHWGRIASGDRFLADPNEAQRLREVF-AADAVEMEGAAALWTAGRLGVPMALVRAISDGAGDEATVNFQEFLEVASRR
             Thermus  AFGVAFFPSDPALLARAEKAALA--LGLPFRRGVVATGDRFLAQREEAERLRALH-GADAVEMEGAAALMVAWRFRHPMVLLRVVTDGAGEGAALDFQAFLREAARRLGLLARALVE
        T.islandicus  ALGVRFFPSDPALLARVEEAAGR--LGLPHRLGVVATGDRFLADPKEAQGLRERF-GAQAVEMEGAAALMVAFRFRHPMALVRAVTDGAGEGAALDFQAFLREASRRLGLLARALV
       Nitratiruptor  EGKVY-VESDPVLREIAKDVAKQ--MQVPLKEGTIATGDQFIADPKRKEWIQKTF-DADALEMEGAAVAVVCDAFDIPFFILRSISDAADMDASFDFDTFLKSSSKRSAEFILKMVDAIIQ
        Caminibacter  ESEVY-FECDKSLNSIAKSVAKK--LNLKLKEGIIASGDQFIHSKEKKEWIKKTF-NASAIEMEGGAVGCVCFTLNIPFFMLRAISDSAEEGAGVDFDEFLEESSKVSAKFLIEMLKE
        Anoxybacillus  GLPAR-YVSDEKLVAIAEKSAAN-IRGVQVAKGLIATGDSFMHDPARVEFVRTQFPELCAVEMEAAAIAQVCHQFGVPFVVIRALSDIAGKESNVSFEQFLHKAALHSSELVQAMVNEL
      A.amylolyticus  GLPAR-YVSDEKLVAIAEKSAAN-IRGVQVAKGLIATGDSFMHDPARVEFVRTQFPELCAVEMEAAAIAQVCHQFGVPFVVIRALSDIAGKESNVSFEQFLHKAALHSSELVQAMVNEL
         A.thermarum  GMPAR-YKADERLVQAAETSAAH-IRDIQVAKGLIATGDSFMNDPARVDFVRTQFPDLYAVEMEAAAIAQVCHQFGVPFVVIRALSDIAGKESNVSFEQFLQKAALHSSELVQWMVNEL
      A.flavithermus  GMPAR-YAADERLIQAAETSAAH-IRDIQVAKGLIATGDSFMHDPARVDFVRTQFPDLYAVEMEAAAIAQVCHQFNVPFVVIRALSDIAGKESNVSFEQFLQKAALHSSELVQLMVNELF--VRTQFPDL
    A.suryakundensis  GMPAR-YVADERLVQAAEASAAH-IRDIQVAKGLIATGDSFMHDPARVDFVRTQFPDLYAVEMEAAAIAQVCHQFAVPFVVIRALSDIAGKESNVSFEQFLQKAALHSSELVQLMVNEL
      A.geothermalis  GMPAR-YAADAKLVAIAEASAER-IQEIQVVKGLIATGDSFMNDPERVAFVREKLTDLHAVEMEGAAIAQVCFQFGVPFVVIRALSDIAGKESNVSFEQFLHKAALHSSELVQMMVDELK--H
      G.subterraneus  GLPAR-YRADETLVEAAKQAAAR-LDGLQAAVGLIATGDSFMNDPKRVEFVRGQFPELCAVEMEAAAIAQVCVQFETPFVIIRALSDIAGEESDVSFEQFLETAAKHSAELVLSMLAVVQ--K
      G.kaustophilus  GLPAR-YRADEALVEAAKQAAAR-LDGLQAVTGLIATGDSFMNDPKRVEFVRGQFPELCAVEMEAAAIAQVCVQFGTPFVIVRALSDIAGEESDVSFEQFLETAAKHSAELVLSML
 G.thermocatenulatus  GLPER-YRADEALVEAAKQAAAR-LDGLQAVTGLIATGDSFMNDPTRVEFVRGQFPELCAVEMEAAAIAQVCVQFGTPFVIIRALSDIAGEESNVSFEQFLETAAKHSAELVLSML
G.caldoxylosilyticus  GMPAR-YQADKTLIDIAKRSAQE-INDVQVVTGLIATGDSFMNDPARVEFVRGKFPELCAVEMEAAAIAQVCTQFAVPFVIIRALSDIAGKESDISFEQFLDTAAKHSARLVASMLSLL
G.stearothermophilus  GMPAR-YQANKTLIDIAKRGAQE-ISDVQVVTGLIATGDSFMNDPERVEFVRGKFPELCAVEMEAAAIAQVCTQFAVPFVIIRALSDIAGKESDISFEQFLDTAAKHSARLVVSMLSLL
G.thermoglucosidasiu  GMPAR-YKADVTLVKIAEQNAKQ-IKDIQVVKGLIATGDSFMSDPARVEFVRSKFPELCAAEMEAAAIAQVCTQFAVPFVIIRALSDIAGKESDVSFEQFLDTAAKHSADLVLSIVSSL
             T.kivui  RMEESIFRADGKLVELAKKTAENIAESD-VYVGRIISGDKFVSSKEEAKRLGEMF-NAYAVEMEGAAIGHVAYLNNIPFVIIRSISDNANDEASVDFSAFVEHAANISSKIVKEMIKLL
     T.thermocopriae  RMEESIFKADEKLVELAKKVAKNIVEGR-AYVGRIISGDKFVSSKEEAKRLGKMF-NAYAVEMEGAAIAHVAFLNSIPFVIIRSISDNANEEASIDFNTFIEQAAKSSTKIVSEMVKLL
   T.saccharolyticum  RMKTSVFKADEYLIDVAYKAANDNIDGK-AYIGRIVSGDKFISSKDEALKLGKLF-NALAVEMEGAAIAHTSYLNNVPFVVIRSISDNADGNAAKDFSQFVKEAAIVSSNIVKEMIN
       T.aotearoense  RMKTSVFKADEYLIDVAYKAANDNIDGK-VYIGRIVSGDKFISSKDEALKLGKLF-NASAVEMEGAAIAHTSYLNNVPFVVIRSISDNADGNAAKDFSQFVKEAAIVSSNIVKEMIN
      T.xylanolyticum  RMKTSVFKADEYLIDVAYKAANDNIDGK-AYIGRIVSGDKFISSKDEALKLGRLF-NALAVEMEGAAIAHTSYLNNIPFVIIRSISDNADGNATKDFSQFVKEAAIVSSNIVKEMIN
T.thermosaccharolyti  RMKTSVFKADKYLIDIALKAANDNIDGK-AYVGRIVSGDRFVSSKEEARRLGQQF-NAYAVEMEGAAIAHTAYLNNIPFVIIRSISDNADGNATSDFNLFVKKASIVSSNLVKEMINMI
            T.oceani  NLGIKAFTADETLVQIAQEVARKQVKGRRAITGRILSGDQFISSKEKVKFLRSFF-DGACVEMEGGAIGQVCYLNKVPFLIIRSISDRADGKAVSVYETFADEAARNSSHIVLGIIKLYEGFVRSKFPDL
          Thermotoga  -GDEGIIVSGSEDLENKVIAYS----NREIKTGLIASGDAFVTTKEKAEEIKRRT-GALCVDMDSAAVAKVCHENEKKFLAIKTIVDVCGRETEEEF
       T.neapolitana  -GDEGIIVSGSEDLEDKVIAYS----NREIKTGLIASGDAFVTVKEKAEEIKRRT-GALCVDMDSAAVAKVCHENEKKFLAIKTIVDICGRETEEEFRKNYERYGFLSNLILLDVLKK
          T.maritima  -GDEGIIVSGSEDLEDKVIAYS----NREIKTGLIASGDAFVTVKEKAEEIKRRT-GALCVDMDSAAVAKVCYENEKKFLAIKTIVDICGRETEEEF
         Thermosipho  -KNVRIFKASDVLMERIYDIFN------DIKFGVIASGDVFVDSEEEKERIYRET-NAEVVDMDSAAIAKVCAENDINFCALKIVVDSSMEGSEIEIKRNLKRLAPFPSAIIAEMLEK
    Fervidobacterium  -YTTLKIESSSKLLEKISDVYD------KINFGPIVSGDVIVSDRETKTKLYEKY-GAFAVDMDSAAIAKVCYQNGVEFCALKVIVDTSEEQAQIEYEQNFRKFSSLPSAIISELLDK
           F.nodosum  -GNIMPIEASNVLLEKIADVYK------YVAFGPMVSGDLIVSDKKMKEMLYDKY-KALAVDMDSAAISKVCKENGVEFCSLKVIVDTSEEQAHTEYEQNFRKFSSLPSTIVSELLDK
           Consensus  ........ad..l.......a...........G.!asGD.F....e.ae.....f..a.a!#M#gAA.a.vc.....pf...r.isD.ag..a...%..fl..a................

                      261     270       280       290       300       310       321
                      |--------+---------+---------+---------+---------+---------+|
         Meiothermus
     M.chliarophilus
          M.silvanus
             Thermus
        T.islandicus
       Nitratiruptor
        Caminibacter
        Anoxybacillus
      A.amylolyticus
         A.thermarum
      A.flavithermus  YAVEMEAAAIAQVCHQFGVPFVVIRALSDIAGKESNVSFEQFLQKAALHSSELVQWMVNEL
    A.suryakundensis
      A.geothermalis
      G.subterraneus
      G.kaustophilus
 G.thermocatenulatus
G.caldoxylosilyticus
G.stearothermophilus
G.thermoglucosidasiu
             T.kivui
     T.thermocopriae
   T.saccharolyticum
       T.aotearoense
      T.xylanolyticum
T.thermosaccharolyti
            T.oceani  CAVEMEAAAIAQVCTQFAVPFVIIRALSDIAGKESDVSFEQFLDTAAKHSADLVLSIVSSL
          Thermotoga
       T.neapolitana
          T.maritima
         Thermosipho
    Fervidobacterium
           F.nodosum
           Consensus
```
