# Supplementary material for: Quorum sensing in thermophiles: prevalence of autoinducer-2 system
Source: BMC Microbiol. 2018 Jun 28;18:62. doi: 10.1186/s12866-018-1204-x (PMC6022435; doi:10.1186/s12866-018-1204-x)
Supplement: Supplementary file 12 — Multiple sequence alignment of RbsB protein from mesophilic and thermophilic eubacteria by MultAlin. Conserved residues are highlighted in red within boxes. (PDF 142 kb) [file 12866_2018_1204_MOESM12_ESM.pdf]

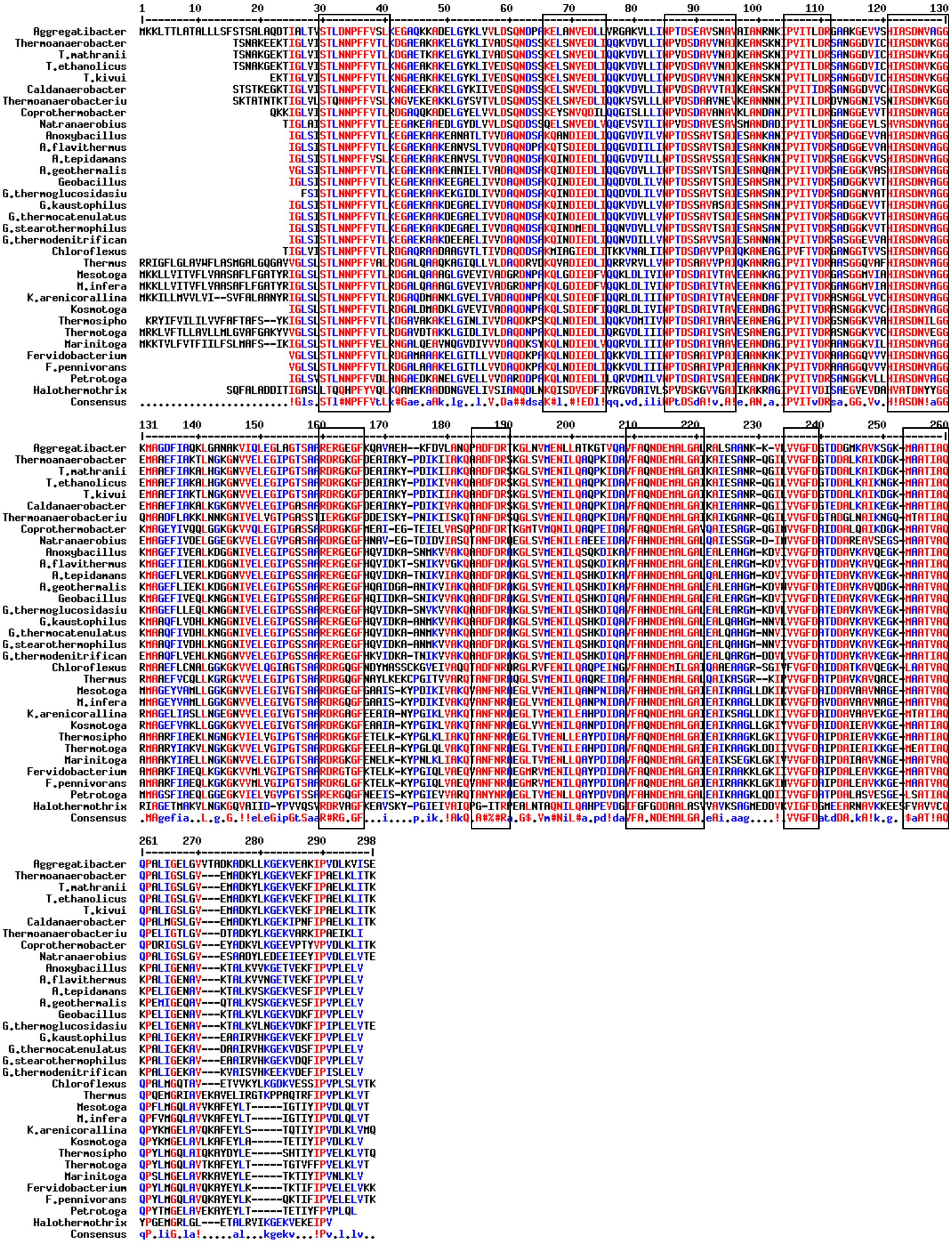
Aggregatibacter
Thermoanaerobacter
T.mathranii
T.ethanolicus
T.kivui
Caldanaerobacter
Thermoanaerobacteriu
Coprothermobacter
Natranaerobius
Anoxybacillus
A.flavithermus
A.tepidamans
A.geothermalis
Geobacillus
G.thermoglucosidasiu
G.kaustophilus
G.thermocatenulatus
G.stearothermophilus
G.thermodenitrifican
Chloroflexus
Thermus
Mesotoga
M.infera
K.arenicorallina
Kosmotoga
Thermosipho
Thermotoga
Marinitoga
Fervidobacterium
F.pennivorans
Petrotoga
Halothermothrix
Consensus
