# Supplementary material for: Quorum sensing in thermophiles: prevalence of autoinducer-2 system
Source: BMC Microbiol. 2018 Jun 28;18:62. doi: 10.1186/s12866-018-1204-x (PMC6022435; doi:10.1186/s12866-018-1204-x)
Supplement: Supplementary file 13 — STRING analysis of LuxS protein of Meiothermus ruber. (PDF 202 kb) [file 12866_2018_1204_MOESM13_ESM.pdf]

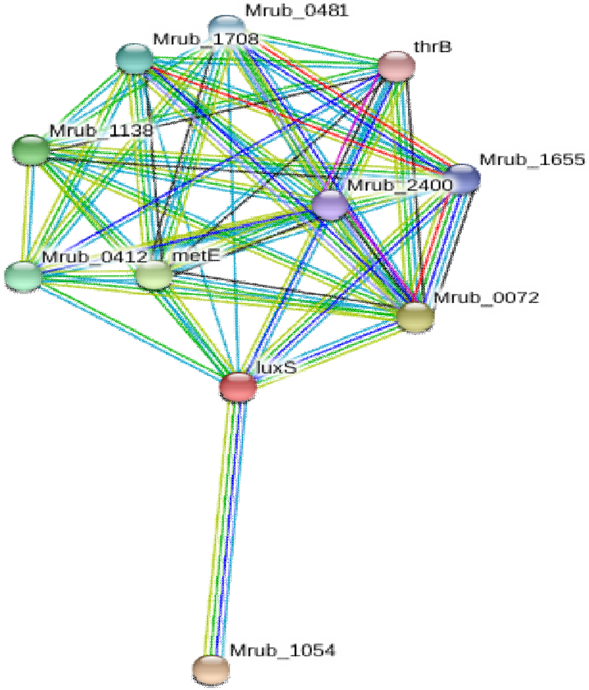

Mrub_1054	*Adenosylhomocysteine nucleosidase (227 aa)*
Mrub_0072	*Cys/Met metabolism pyridoxal-phosphate-dependent protein (368 aa)*
metE	*5-methyltetrahydropteroyltriglutamate– homocysteine methyltransferase;*
Mrub_1138	*methionine synthase (1215 aa)*
Mrub_0412	*aminotransferase class I and II (386 aa)*
Mrub_1708	*O-acetylhomoserine/O-acetylserine sulfhydrylase (430 aa)*
Mrub_0481	*O-acetylhomoserineaminocarboxypropyltransferase (422 aa)*
Mrub_1655	*cysteine synthase (315 aa)*
Mrub_2400	*Homoserine dehydrogenase (338 aa)*
thrB	*homoserine kinase;*
