# Supplementary material for: Quorum sensing in thermophiles: prevalence of autoinducer-2 system
Source: BMC Microbiol. 2018 Jun 28;18:62. doi: 10.1186/s12866-018-1204-x (PMC6022435; doi:10.1186/s12866-018-1204-x)
Supplement: Supplementary file 14 — STRING analysis of LuxS protein of Geobacillus thermoglucosidasius. (PDF 225 kb) [file 12866_2018_1204_MOESM14_ESM.pdf]

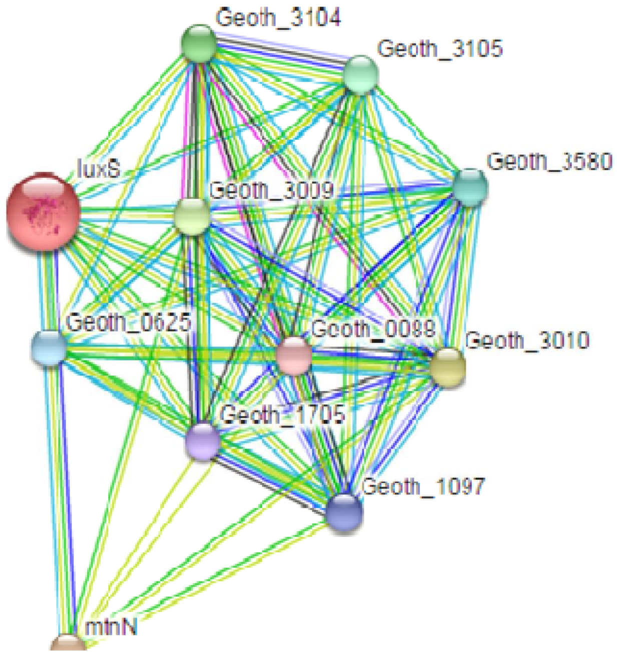

mtnN	*5'-methylthioadenosine/S-adenosylhomocysteine nucleosidase*
Geoth_3010	*Cys/Met metabolism pyridoxal-phosphate-dependent protein (367 aa)*
Geoth_3009	*Cys/Met metabolism pyridoxal-phosphate-dependent protein (392 aa)*
Geoth_3104	*methionine synthase (616 aa)*
Geoth_3105	*methionine synthase (1136 aa)*
Geoth_3580	*O-acetylhomoserine/O-acetylserine sulfhydrylase (436 aa)*
Geoth_0625	*cystathionine beta-lyase (386 aa)*
Geoth_1097	*Homocysteine desulfhydrase (378 aa)*
Geoth_1705	*cysteine synthase (307 aa)*
Geoth_0088	*cysteine synthase (308 aa)*
