# Supplementary material for: Quorum sensing in thermophiles: prevalence of autoinducer-2 system
Source: BMC Microbiol. 2018 Jun 28;18:62. doi: 10.1186/s12866-018-1204-x (PMC6022435; doi:10.1186/s12866-018-1204-x)
Supplement: Supplementary file 15 — STRING analysis of LuxS protein of Caminibacter mediatlanticus. (PDF 90 kb) [file 12866_2018_1204_MOESM15_ESM.pdf]

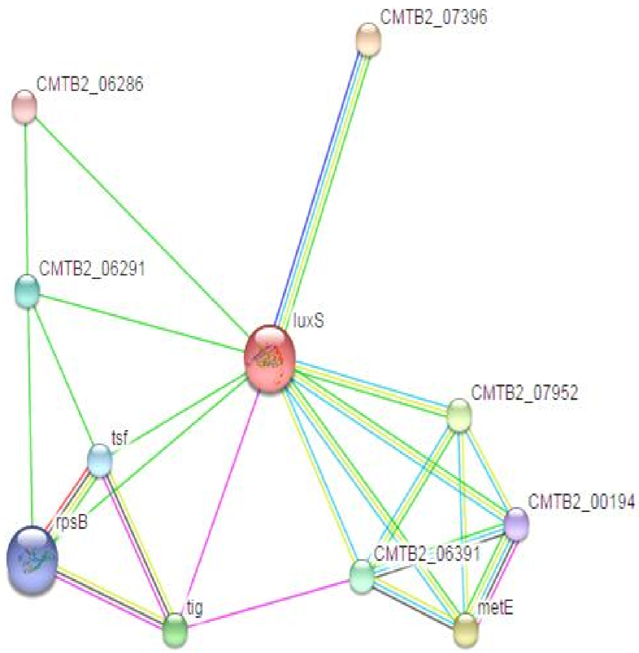

●	CMTB2_07396	*5'-methylthioadenosine/S-adenosylhomocysteine nucleosidase (230 aa)*
●	metE	*Methionine synthase, vitamin-B12 independent isozyme;*
●	CMTB2_07952	*Cysteine synthase (272 aa)*
●	tig	*PPlase; Involved in protein export.*
●	CMTB2_06391	*Homoserine dehydrogenase (417 aa)*
●	CMTB2_06291	*Twin-arginine translocation pathway signal (156 aa)*
●	tsf	*Elongation factor Ts; Associates with the EF-Tu.GDP complex*
●	rpsB	*30S ribosomal protein S2 (274 aa)*
●	CMTB2_00194	*Methylenetetrahydrofolate reductase (296 aa)*
●	CMTB2_06286	*Putative transmembrane magnesium and cobalt transporter protein (310 aa)*
