# Supplementary material for: Quorum sensing in thermophiles: prevalence of autoinducer-2 system
Source: BMC Microbiol. 2018 Jun 28;18:62. doi: 10.1186/s12866-018-1204-x (PMC6022435; doi:10.1186/s12866-018-1204-x)
Supplement: Supplementary file 16 — NCBI accession number of genomes of thermophililc archaea used in the study. (PDF 24 kb) [file 12866_2018_1204_MOESM16_ESM.pdf]

**Archaea**	**Accession numbers**
Thermococcus barophilus	NC_014804
Thermococcus celer	NZ_CP014854
Thermococcus chitonophagus	NZ_CP015193
Thermococcus gammatolerans	NC_012804
Thermococcus kodakarensis	NC_006624
Thermococcus litoralis	NC_022084
Thermococcus profundus	NZ_CP014862
Pyrococcus abyssi	NC_000868
Pyrococcus furiosus	NC_003413
Pyrococcus horikoshii	NC_000961
Picrophilus torridus	NC_005877
Pyrolobus fumarii	NC_015931
Pyrodictium abyssi	WP_048147334
Metallosphaera sedula	NC_009440
Aeropyrum pernix	BA000002
Nanoarchaeum equitans	AE017199
