# Supplementary material for: Quorum sensing in thermophiles: prevalence of autoinducer-2 system
Source: BMC Microbiol. 2018 Jun 28;18:62. doi: 10.1186/s12866-018-1204-x (PMC6022435; doi:10.1186/s12866-018-1204-x)
Supplement: Supplementary file 17 — NCBI accession number of genomes of thermophililc bacteria used in the study. (PDF 137 kb) [file 12866_2018_1204_MOESM17_ESM.pdf]

Bacteria	Accession number
Desulfurobacterium thermolithotrophum	NC_015185
Desulfurobacterium atlanticum	WGS: NZ_FZOB00000000
Sulfurihydrogenibium yellowstonense	WGS: NZ_ABZS00000000, SAH hydrolase: EEP59824
Sulfurihydrogenibium azorense	NC_012438
Sulfurihydrogenibium subterraneum	WGS: NZ_JHUV00000000, SAH hydrolase: WP_028950408
Thermovibrio ammonificans	NC_014926
Anaerophaga thermohalophila	WGS: NZ_AEWI00000000, SAH hydrolase: WP_044107885, RbsB: WP_016776256
Chloroflexus aurantiacus	NC_010175
Chloroflexus aggregans	NC_011831
Thermomicrobium roseum	NC_011959
Deferribacter desulfuricans	NC_013939
Marinithermus hydrothermalis	NC_015387
Meiothermus ruber	NC_013946
Meiothermus silvanus	NC_014212
Meiothermus chliarophilus	WGS: NZ_AUQW00000000, LuxS: WP_027892296, Pfs: WP_027892298, RbsB: WP_051303839, SAH hydrolase: WP_051304219
Meiothermus taiwanesis	WP_027888138
Meiothermus cerbereus	WGS: NZ_JHVI00000000, LuxS: WP_027878194
Meiothermus rufus	WGS: NZ_AUHY00000000, LuxS: WP_027883135
Oceanithermus profundus	NC_014761
Thermus filiformis	WGS: NZ_JPSL00000000, LuxS: WP_038063204, Pfs: WP_027892298
Thermus parvatiensis	NZ_CP014141
Thermus caliditerrae	WGS: NZ_JQNC00000000, LuxS: WP_038047518, Pfs: WP_038047516
Thermus oshimai	NC_019386
Thermus igniterrae	WGS: NZ_AQWU00000000, LuxS: WP_018110943, Pfs: WP_018110942
Thermus scotoductus	NC_014974
Thermus thermophilus	NC_006461
Thermus amyloliquefaciens	WGS: NZ_JQMV00000000, LuxS: WP_038057759, Pfs: WP_038057757
Thermus islandicus	WGS: NZ_ATXJ00000000, LuxS: WP_022797829, Pfs: WP_022797830, RbsB: WP_022799484

Thermus aquaticus	NZ_CP010822
Dictyoglomus thermophilum	NC_011297
Ammonifex degensii	NC_013385
Anoxybacillus geothermalis	WGS: NZ_JYCG00000000, LuxS: WP_044745499, Pfs: WP_044744701, LsrB: WP_044746354, RbsB: WP_044741598
Anoxybacillus thermarum	WGS: NZ_JXTH00000000, LuxS: WP_043967964, Pfs: WP_043966005
Anoxybacilllus amylolyticus	NZ_CP015438
Anoxybacillus suryakundensis	NZ_LIOK00000000, LuxS: WP_055440163, Pfs: WP_055441930
Anoxybacillus flavithermus	NC_011567
Caldanaerobacter subterraneus	NC_003869
Caldicellulosiruptor saccharolyticus	NC_009437
Caldicellulosiruptor kronotskyensis	NC_014720
Caldicellulosiruptor bescii	NC_012034
Carboxydothermus hydrogenoformans	NC_007503
Carboxydothermus ferrireducens	WGS: NZ_ATYG00000000, SAH hydrolase: WP_028053005
Coprothermobacter proteolyticus	NC_011295
Coprothermobacter platensis	WGS: NZ_ARJK00000000, SAH hydrolase: WP_018962483
Geobacillus caldoxylosilyticus	WGS: NZ_BAWO00000000, LuxS: WP_017434878, Pfs: WP_042406345
Geobacillus stearothermophilus	NZ_CP008934
Geobacillus thermoglucosidasius	NZ_CP012712
Geobacillus kaustophilus	NC_006510
Geobacillus subterraneus	NZ_CP014342
Geobacillus icigianus	WGS: NZ_JPYA00000000, LuxS: WP_033018295, Pfs: WP_033018027, RbsB: WP_033021787
Geobacillus thermoleovorans	NZ_CP014335
Geobacillus thermodenitrificans	NC_0093281
Halothermothrix orenii	NC_011899
Moorella thermoacetica	NC_007644
Natranaerobius thermophilus	NC_010718
Pelotomaculum thermopropionicum	AP009389
Thermosediminibacter oceani	NC_014377
Thermoanaerobacter kivui	NZ_CP009170

Thermoanaerobacter thermocopriae	WGS: NZ_JADV00000000, LuxS: WP_028992173, Pfs: WP_028992157, RbsB: KUJ90334
Thermoanaerobacterium thermosaccharolyticum	NC_014410.1
Thermoanaerobacterium saccharolyticum	CP003184.1
Thermoanaerobacterium aotearoense	WGS: NZ_AYSN00000000, LuxS: WP_014759399, Pfs: WP_014758871
Thermoanaerobacterium xylanolyticum	NC_015555.1
Thermosinus carboxydivorans	WGS: NZ_AAWL00000000, SAH hydrolase: WP_007288137
Thermodesulfovibrio aggregans	WGS: NZ_BCNO00000000, SAH hydrolase: WP_059176038
Thermodesulfovibrio thiophilus	WGS: NZ_AUIU00000000, SAH hydrolase: WP_028844893
Caminibacter mediatlanticus	WGS: NZ_ABCJ00000000, LuxS: WP_007475079, Pfs: EDM24061
Desulfacinum infernurn	WGS: NZ_FQVB00000000
Hippea maritima	NC_015318
Hippea jasoniae	WGS: NZ_JQLX00000000, SAH hydrolase: WP_035587885
Hippea alviniae	NZ_ATUV00000000, SAH hydrolase: WP_022670137.1
Nitratiruptor SB155-S	NC_009662
Fervidobacterium islandicum	NZ_CP014334
Fervidobacterium gondwanense	WGS: NZ_FRDJ00000000
Fervidobacterium changbaicum	WGS: NZ_FNDL00000000
Fervidobacterium nodosum	NC_009718
Kosmotoga pacifica	NZ_CP011232
Kosmotoga olearia	NC_012785
Kosmotoga arenicorallina	WGS: NZ_JFHK00000000, RbsB: OAA27217
Marinitoga piezophila	NC_016751
Marinitoga hydrogenitolerans	WGS: NZ_JFHK00000000
Petrotoga mobilis	NC_010003
Petrotoga mexicana	WGS: NZ_AZRN00000000
Petrotoga halophila	WGS: NZ_JALY00000000
Thermodesulfatator indicus	NC_015681
Thermodesulfatator atlanticus	WGS: NZ_ATXH00000000, SAH hydrolase: WP_022852355.1
Thermodesulfatator autotrophicus	WGS: NZ_LSFI00000000, SAH hydrolase: WP_068543003.1
Thermosipho africanus	NC_011653
Thermosipho melanesiensis	NC_009616

Thermotoga petrophila	NC_009486
Thermotoga maritima	NC_000853
Thermotoga neopolitana	NC_011978
Thermotoga naphthophila	NC_013642
